# Cyanobacterial Toxins and Peptides in Lake Vegoritis, Greece

**DOI:** 10.3390/toxins13060394

**Published:** 2021-06-01

**Authors:** Sevasti-Kiriaki Zervou, Kimon Moschandreou, Aikaterina Paraskevopoulou, Christophoros Christophoridis, Elpida Grigoriadou, Triantafyllos Kaloudis, Theodoros M. Triantis, Vasiliki Tsiaoussi, Anastasia Hiskia

**Affiliations:** 1 Laboratory of Photo-Catalytic Processes and Environmental Chemistry, Institute of Nanoscience & Nanotechnology, National Center for Scientific Research “Demokritos”, Patriarchou Grigoriou E & 27 Neapoleos Str, 15310 Agia Paraskevi, Athens, Greece; s.zervou@inn.demokritos.gr (S.-K.Z.); k.paraskevopoulou@inn.demokritos.gr (A.P.); c.christoforidis@inn.demokritos.gr (C.C.);t.kaloudis@inn.demokritos.gr (T.K.); t.triantis@inn.demokritos.gr (T.M.T.); 2 The Goulandris Natural History Museum—Greek Biotope/Wetland Centre, 14th km Thessaloniki-Mihaniona, Thermi P.O. Box 60394, 57001 Thessaloniki, Greece; kmosch@ekby.gr (K.M.); vasso@ekby.gr (V.T.); 3 Water Resources Management Agency of West Macedonia, 50100 Kozani, Decentralized Administration of Epirus—Western Macedonia, Greece; grig.elpida@gmail.com

**Keywords:** cyanotoxins, microcystins, cylindrospermopsin, cyanopeptides, anabaenopeptins, microginins, aeruginosins, aeruginosamide, SPE, LC-MS/MS, Lake Vegoritis

## Abstract

Cyanotoxins (CTs) produced by cyanobacteria in surface freshwater are a major threat for public health and aquatic ecosystems. Cyanobacteria can also produce a wide variety of other understudied bioactive metabolites such as oligopeptides microginins (MGs), aeruginosins (AERs), aeruginosamides (AEGs) and anabaenopeptins (APs). This study reports on the co-occurrence of CTs and cyanopeptides (CPs) in Lake Vegoritis, Greece and presents their variant-specific profiles obtained during 3-years of monitoring (2018–2020). Fifteen CTs (cylindrospermopsin (CYN), anatoxin (ATX), nodularin (NOD), and 12 microcystins (MCs)) and ten CPs (3 APs, 4 MGs, 2 AERs and aeruginosamide (AEG A)) were targeted using an extended and validated LC-MS/MS protocol for the simultaneous determination of multi-class CTs and CPs. Results showed the presence of MCs (MC-LR, MC-RR, MC-YR, dmMC-LR, dmMC-RR, MC-HtyR, and MC-HilR) and CYN at concentrations of <1 μg/L, with MC-LR (79%) and CYN (71%) being the most frequently occurring. Anabaenopeptins B (AP B) and F (AP F) were detected in almost all samples and microginin T1 (MG T1) was the most abundant CP, reaching 47.0 μg/L. This is the first report of the co-occurrence of CTs and CPs in Lake Vegoritis, which is used for irrigation, fishing and recreational activities. The findings support the need for further investigations of the occurrence of CTs and the less studied cyanobacterial metabolites in lakes, to promote risk assessment with relevance to human exposure.

## 1. Introduction

Cyanobacteria are common photosynthetic microorganisms found in lakes and surface water reservoirs, which can, under favorable conditions, grow massively to form blooms [1]. Several cyanobacteria species produce potent toxic compounds as secondary metabolites, called cyanotoxins (CTs) [2,3]. Several incidents of wild and domestic animal poisoning as well as human health effects due to toxic cyanobacterial blooms have been reported [4,5,6,7].

Cyanotoxins comprise a large number of compounds presenting a variety of chemical structures (Figure S1). Microcystins (MCs) [8] and nodularins (NODs) [9] are cyclic peptides typically characterized by the presence of the unique amino acid Adda ((2S,3S,8S,9S)-3-amino-9-methoxy-2,6,8-trimethyl-10-phenyl deca-4,6-dienoic acid) in their structure, which is associated with their hepatotoxicity [2,10,11]. The alkaloid cylindrospermopsin (CYN) is cytotoxic, dermatotoxic, hepatotoxic, and possibly carcinogenic [12,13]. Anatoxin-a (ATX) is a bicyclic secondary amine (2-acetyl-9-azabicyclo (1,2,4) non-2-ene) with acute neurotoxicity [14].

In addition to CTs, cyanobacteria can also produce a wide variety of other metabolites, including compounds of peptide structure such as microginins (MGs), aeruginosins (AERs), aeruginosamides (AEGs), and anabaenopeptins (APs) [15] (Figure S2). MGs are a group of linear oligopeptides characterized by the presence of a decanoic acid derivative, 3-amino-2-hydroxy-decanoic acid (Ahda) at the *N*-terminus [16]. Although not fully investigated, MGs were shown to present strong protease inhibition with MG variants displaying ecotoxicological effects [17]. AERs are linear peptides that include both a derivative of hydroxyl-phenyl lactic acid (Hpla) at the *N*-terminus, the amino acid 2-carboxy-6-hydroxyoctahydroindole (Choi) and an arginine derivative at the C-terminus [18]. Studies on their bioactivity revealed that they inhibit serine proteases trypsin and thrombin, while AER 828A was found to be toxic to *Thamnocephalus platyurus* [19]. The linear peptides AEGs, characterized by the presence of prenyl and thiazole groups, are an understudied group of cyanobacterial metabolites for which limited knowledge is available with regards to their occurrence in cyanobacterial blooms and their bioactivity [20,21]. APs are cyclic peptides with the general structure of X1-CO-[Lys-X3-X4-MeX5-X6]. Lysine (Lys) is present in all variants while X1, X3, X4, X5 and X6 are variable amino acids. A side chain of one amino acid is attached to the ring through an ureido bond with Lys [15]. Recently, it has been reported that APs can be very abundant in nature [22,23]. To date, little is known about the potential health effects of APs on animals and humans [17]. Anabaenopeptin F (AP F) is considered a protease inhibitor and it was shown to inhibit protein phosphatases similarly to MCs [24]. Additionally, Anabaenopeptin B (AP B) and AP F were shown to induce lysis of the cyanobacteria *Microcystis aeruginosa* that can drastically influence cyanobacterial community dynamics and trigger the release of toxins into surface waters [25].

Toxic cyanobacteria blooms occur worldwide [26], with climate predictions suggesting their increase in the future in terms of frequency and severity [27,28,29]. Therefore, there is an urgent need to monitor toxic cyanobacteria and their toxins, especially in water bodies intended to be used as drinking water supplies or for recreational activities, particularly by children [30]. At the same time, there is need to better assess the occurrence, bioactivity, and effects of other cyanobacterial peptides (CPs) in order to improve risk assessment and the development of management strategies for cyanobacterial blooms.

Lake Vegoritis is a large natural lake covering an area of 60 km^2^ in the region of Western Macedonia, in north-western Greece. The banks of the lake are an ideal refuge for many wild birds and it has remarkable fish fauna, which includes a large variety of species. The lake’s sensitive ecosystem belongs to the European Network of Protected Areas (NATURA 2000), due to its important habitats and rich biodiversity. A part of its littoral zone was also designated as a bathing area, according to Directive 2006/7/EC. The ecological and historical background of toxic cyanobacterial blooms of Lake Vegoritis, as well as the recreational activities that it offers, create a growing concern about the possible effect of CTs and CPs to its ecosystem and human health. Besides its ecological importance, the lake is used for irrigation, fishing, and recreational activities.

Recently, a multi-lake survey covering 14 lakes in Greece was conducted with the aim to assess the presence of a wide range of CTs from different classes including MCs, NODs, CYN and ATX, using liquid chromatography coupled to tandem mass spectrometry (LC-MS/MS) [31]. In the frame of that study, it was found that water from Lake Vegoritis contained MCs and traces of CYN. Although the occurrence of CTs in Lake Vegoritis was confirmed and documented, it was based only on individual samples and the study was not designed to provide information concerning the spatial and temporal variation of CTs. Furthermore, there is complete lack of information regarding the co-occurrence of other cyanobacterial peptides such as MGs, AERs, AEGs, and APs.

In response to the above study, a 3-year monitoring program of Lake Vegoritis was initiated in 2018, with the aim to characterize the cyanobacterial species present and to assess the occurrence of various classes of CTs and CPs in the lake. Fifteen CTs (i.e., CYN, ATX, NOD and dmMC-RR, MC-RR, MC-YR, MC-HtyR, dmMC-LR, MC-LR, MC-HilR, MC-WR, MC-LA, MC-LY, MC-LW, and MC-LF) and ten CPs (i.e., MG FR1, MG FR3 MG T1, MG T2, AER 602/K139, AER 298A, AEG A, AP B, AP F and oscillamide (OSC Y)) were targeted. Selection of the targeted cyanobacterial metabolites was based on their frequency of detection in other Greek lakes [31,32]. To implement this monitoring program, a new method for simultaneous determination of various classes of CPs in addition to CTs was developed and validated, extending a previously validated LC-MS/MS analytical protocol [31,33] to include MGs, AERs, AEGs and APs. Using this new protocol, the detection of several classes of CTs and CPs would be possible in a single analytical run.

Results obtained by this 3-year study enable risk assessment and management of toxic cyanobacterial blooms by the lake’s authorities. The study’s findings also facilitate the reliable and effective communication of the risks to the general public and stakeholders, with regards to the uses of the lake, such as irrigation, fishing, and recreational activities.

## 2. Results and Discussion

### 2.1. Physico-Chemical Parameters of Lake Vegoritis

Lake Vegoritis is under pressure from point source and diffuse pollution. As reported by monitoring results from 2012–2015, the lake was in moderate ecological and good chemical status [34]. The concentrations of most of the physicochemical quality parameters, with the exception of nitrates and sulfates, did not fluctuate considerably during the study period (Table S1). The F^−^, NO_2_^−^, Br^−^ and PO_4_^3−^ ions were measured below the method’s limit of quantification (LOQ) during the whole study period. The Cl^−^ ranged between 32–40 mg/L, with a single high measurement (58 mg/L) in July 2020. The NO_3_^−^ ions ranged from below LOQ to 1.0 mg/L, with the highest values detected in the winter of 2018 and 2020 and in August 2020. The greatest variability was noticed for SO_4_^2−^ levels that ranged between 84 and 200 mg/L, with no seasonal or other temporal pattern observed. The cation (Na^+^, K^+^, Mg^2+^, Ca^2+^) concentrations measured showed relative stability throughout the study period and no temporal distribution pattern.

In 2018, the highest levels of total phosphorus (TP) were measured. Concentrations ranged from 22 to 60 μg/L and displayed the highest values in February and April (60 and 50 μg/L, respectively). In June 2018, TP decreased to 43 μg/L, while in July 2018 the mean value of the TP concentration was 38 μg/L. Concentrations declined in the following months (<29 μg/L). The following years, 2019 and 2020, TP concentrations were measured at slightly lower levels, 28–50 μg/L and 16–38 μg/L, respectively.

The transparency of the water was measured using the Secchi disc and in June 2018 there was a significant reduction to 0.3 m, from 6.5 m and 6.0 m measured in February 2018 and April 2018, respectively. Such a low value was measured for the first time in the lake throughout the operation of the National Monitoring Water Network (2012–2018). Since then, the transparency of the water in the lake, including bathing area sampling points, presented a noteworthy increase (2.4 m on 4 July 2018 and 3.0 m on 12 July 2017 and 17 July 2018). The Secchi disc was visible to the bottom of the bathing area stations until the autumn. The following years 2019–2020, transparency of the water at the NMWN (National Monitoring Water Network) sampling point ranged from 1.6 m to 4.8 m.

The total suspended solids (TSS) exhibited a high value of 8.46 mg/L in July 2018. During the summer, their concentrations in the lake decreased both at the NMWN sampling point and the bathing area (Site 1). In the next two years, TSS did not exceed 2.54 mg/L, except for two cases in June 2019 (3.75 mg/L) and in September 2020 (5.67 mg/L).

### 2.2. Chlorophyll α

The concentration of chlorophyll α in Lake Vegoritis in June 2018 was high (15.9 μg/L), with a decreasing trend during the following summer months at both the NMWN sampling point and the bathing area (Site 1) (3.0–8.0 μg/L). This reduction was in line with the improvement in water transparency values observed during the same period. Similar values of chlorophyll α concentration (4.2–7.7 μg/L) were measured during the following years, 2019 and 2020. In two samples collected on 10 September 2019 and 22 September 2020 much higher values (19.4 and 31.8 μg/L, respectively) were measured, but no discoloration of water was observed (Table S1).

### 2.3. Phytoplankton

During June 2018, the total phytoplankton biomass was estimated at 2.3 mg/L. The most abundant taxa were the green alga *Sphaerocystis schroeteri* (39,889,429 cells/L), cyanobacteria of the genus *Dolichospermum* (14,864,850 cells/L) and the species *Aphanizomenon flos-aquae* (3,383,107 cells/L). Chlorophyta and cyanobacteria comprised 88% of the biomass. In the subsequent samplings, no bloom, mat or scum (as defined in Directive 2006/7/EC) [35] were visually observed. Furthermore, phytoplankton biomass in samples from NMWN gradually decreased, mainly due to the decrease in the biomass of chlorophytes and dinophytes. The *Dolichospermum* biomass also declined sharply (from 0.75 mg/L in June 2018 to 0.02 mg/L in July 2018 and 0.04 mg/L in August 2018).

At the same period, summer 2018, in the samples from the bathing area the cyanobacteria *Microcystis* spp., *Aphanocapsa* spp., *Dolichospermum* spp., and *Aphanizomenon flos-aquae* were dominant. However, their biomass did not exceed 0.7 mg/L (*Aphanizomenon flos-aquae* in July 2018). The biomass values of the dominant cyanobacteria were measured at higher levels in July (1.6 mg/L), followed by sharp decline in the next months. Regarding the chlorophyte *Sphaerocystis schroeteri*, its biomass was measured at lower levels compared to the measurement of June 2018 at the NMWN sampling point and showed a gradual further decrease during August and September.

During the warm period of 2019 the cyanobacteria biomass (four samples from NMWN sampling point) displayed the opposite trend. Biomass gradually increased during summer until the maximum value of 5.4 mg/L (September 2019), where *Aphanizomenon* spp. biomass was estimated at 2.1 mg/L, *Lemmermanniella* spp. at 2.1 mg/L, and *Raphidiopsis raciborskii* at 0.5 mg/L. In all the other three samples of 2019, *Aphanizomenon* spp. and *Dolichospermum* spp. were the main representatives, though with lower biomass values (up to 0.82 mg/L).

No specific trend was observed during the warm period of 2020. In June 2020 the biomass of cyanobacteria was minimal (0.1 mg/L). Over the next three months it increased up to 1.8 mg/L, as measured in August 2020. In July a *Dolichospermum* species dominated (1.1 mg/L), but low biomass values were measured for *Aphanocapsa* cf. *holsatica* and *Cyanodictyon* species (up to 0.2 mg/L). In August and September, species of the genus *Aphanizomenon* prevailed (1.6 and 0.9 mg/L, respectively). Low biomass values of *Microcystis* species (0.1 and 0.3 mg/L) were also measured. Total phytoplankton and cyanobacteria biomass concentrations measured during the study period are given in Table S1.

### 2.4. Occurrence of Cyanotoxins (CTs) in Lake Vegoritis

A range of CTs (extracellular and intracellular fractions), including CYN, ATX, dmMC-RR, MC-RR, NOD, MC-YR, MC-HtyR, dmMC-LR, MC-LR, MC-HilR, MC-WR, MC-LA, MC-LY, MC-LW, and MC-LF were determined by LC-MS/MS in samples taken during the study (2018–2020). Results are presented in Tables S2 and S3.

In 2018, 14 samples were analyzed from July to November, seven from each one of the sampling sites. None of these samples were found to contain detectable amounts of CYN, ATX and NOD. Τhe analysis of filtered water showed the presence of extracellular MCs, i.e., MC-LR, MC-RR and MC-YR, at concentrations up to 0.029, 0.023 and 0.014 μg/L, respectively. Intracellular (cell-bound) MCs were also detected, including MC-RR, MC-LR, MC-YR, and dmMC-RR at concentrations of up to 0.074, 0.055, 0.026 and 0.003 μg/L, respectively.

During 2019, a total of 24 samples from the two sampling sites were analyzed from April to October. ATX and NOD were not detected in any of the samples. Contrary to findings of 2018 monitoring, CYN was detected mainly in the intracellular (cell-bound) fraction, at concentrations ranging from 0.032 to 0.685 μg/L, and at a lower level in the extracellular (dissolved) fraction. MCs were also detected, with MC-LR and dmMC-LR up to 0.233 and 0.080 μg/L (extracellular fraction) and MC-RR, dmMC-LR, MC-LR, and MC-HilR up to 0.029, 0.055, 0.241, and 0.027 μg/L, respectively (intracellular fraction).

In 2020, 20 samples were analyzed from May to October. Co-occurrence of CYN and MCs was again observed with CYN present during this period at concentrations reaching 0.128 and 0.075 μg/L in extra- and intracellular fractions, respectively. MC-RR, MC-HtyR, dmMC-LR, and MC-HilR (extracellular fraction) were also present at concentrations of up to 0.315 μg/L (22 June 2020, Site 2), while dmMC-RR, MC-RR, MC-YR, MC-HtyR, dmMC-LR, MC-LR, and MC-HilR (intracellular fraction) were up to 0.674 μg/L (7 September 2020, Site 1).

**Figure 1:**
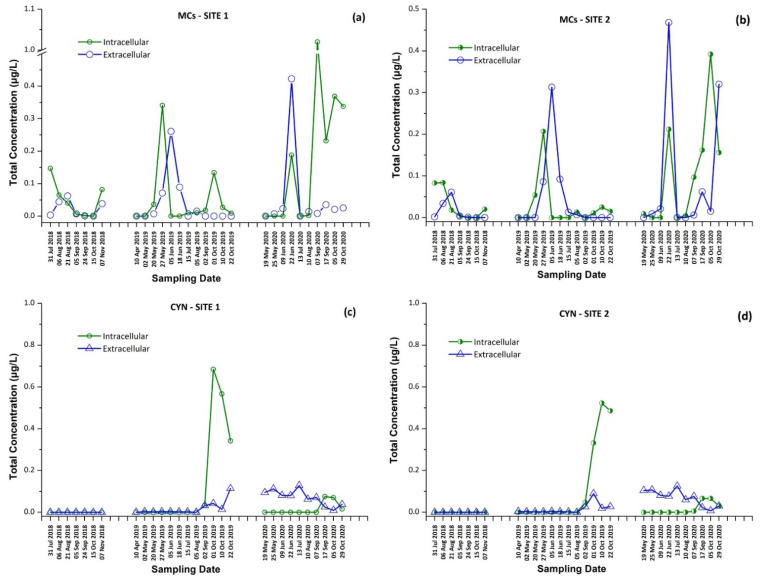
Intracellular and extracellular fractions of microcystins (MCs) and cylindrospermopsin (CYN) per sampling date; MCs at (**a**) Site 1 and (**b**) Site 2, and CYN at (**c**) Site 1 and (**d**) Site 2.

**Figure 2:**
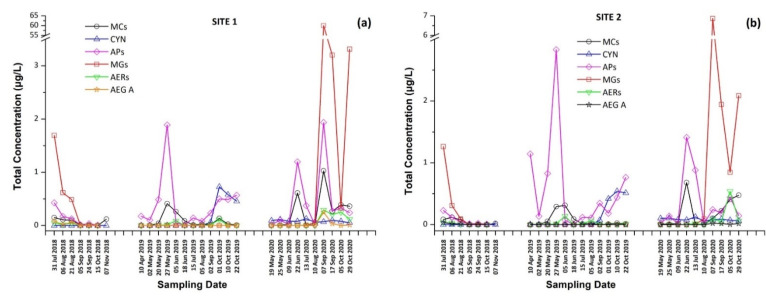
Total concentration (sum of intracellular and extracellular) of cyanotoxins (CTs) and cyanopeptides (CPs) detected per sampling date at (**a**) Site 1 and (**b**) Site 2.

**Figure 3:**
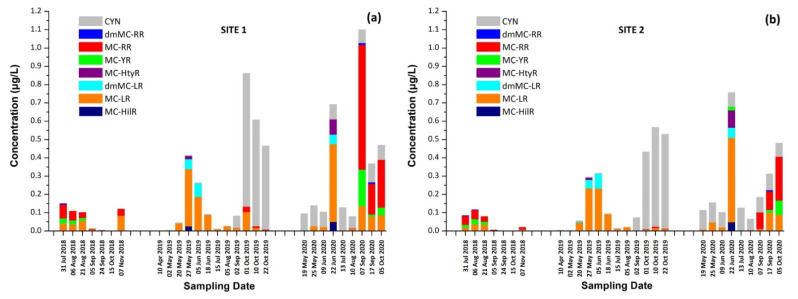
Occurrence of CTs in Lake Vegoritis at (**a**) Site 1 and (**b**) Site 2.

The intracellular and extracellular fractions of MCs and CYN, as well as the total concentrations (sum of intracellular and extracellular) per sampling date and site, are presented in Figure 1 and Figure 2. The occurrence of individual CTs in Lake Vegoritis are shown in Figure 3.

Results for both sampling sites presented a similar seasonal trend in CT concentrations, with an increase during the summer followed by a second outbreak in the autumn. As expected, intracellular MCs tended to appear first, followed by extracellular MCs, due to the release of cell-bound MCs into water after the lysis of cyanobacterial cells, with the exception of June 2020 (Figure 1a,b). The maximum of total MCs, 1.02 μg/L, consisted of dmMC-RR, MC-RR, MC-YR, and MC-LR, and was measured on 7 September 2020 at Site 1, with MC-RR being the most abundant MC variant. The percentage of samples in which each CT was detected is shown in Table 1. MC-LR was detected in 79% of samples through the entire monitoring program, while MC-RR was detected in 50% of samples and was largely absent during 2019.

CYN was also detected in 71% of samples of Lake Vegoritis, while detections occurred only during 2019 and 2020, and not in samples taken in 2018. CYN was found in both extra- and intracellular fractions. The maximum concentration of total CYN was 0.727 μg/L at Site 1 on 1 September 2019 (Figure 1c).

The presence of MCs could be related to the dominant cyanobacteria *Dolichospermum* spp. (*Anabaena* spp.), *Aphanizomenon* spp., and *Microcystis* spp. that were identified in Lake Vegorits during the study period [36]. The presence of CYN may also be attributed to the cyanobacteria *Aphanizomenon* spp. and *Dolichospermum* spp. as well as to *Raphidiopsis raciborskii* [12,37]. These results concur with a previous study where Lake Vegoritis was found to be mainly dominated by *Dolichospermum* spp., *Aphanizomenon* spp., and *Microcystis* spp. [31]. In the same study, CYN, MC-RR and MC-LR were identified in one sample of biomass from Lake Vegoritis (September 2008) at concentrations of <LOQ, 0.118 and 0.049 μg/L, respectively. In a water sample (July 2014), extracellular dmMC-RR, MC-RR, MC-YR, dmMC-LR, MC-LR, and MC-LY (MC-RR: 104 μg/L and MC-LR 96.3 μg/L) were also detected [31]. Although the findings were based on only two samples, the toxin profile (MC-RR, MC-LR) is in agreement with the present study. However, the concentrations reported in the previous study were far higher, possibly because the sampling was targeted rather than systematic, aiming at localized bloom formations.

While there are no recreational beach monitoring programs for toxins in Greece, this is the first study to investigate the presence, the concentration, and the diversity of CTs in a popular lake beach of North Greece devoted to recreational activities. In all cases, the measured concentration of CTs did not exceed the provisional guideline values proposed for recreational water by the World Health Organization (WHO) that have been recently updated and were set at 24 and 6 μg/L for MC-LR and CYN, respectively [38,39].

### 2.5. Detection, Identification and Occurrence of Cyanobacterial Peptides (CPs) in Lake Vegoritis

Although the occurrence of MCs in fresh water bodies is well documented due to the development of analytical protocols [1,31,33,40], data bases [41,42], and a number of commercially available standards, less is known regarding the presence of other CPs. Recent studies showed the presence of CPs in water bodies [17,43], but analysis was mainly done in cyanobacterial biomass, not in water samples. Analytical protocols have not been developed and validated for water samples (extracellular–intracellular fractions), to include cleanup and pre-concentration steps [22]. In this study, we present method performance and validation results for targeted LC-MS/MS analysis of water samples for CPs, based on an analytical workflow previously used for analysis of CTs [33]. The targeted CPs were MG FR1, MG FR3 MG T1, MG T2, AER 602/K139, AER 298A, AEG A, AP B, AP F and OSC Y. The validated method was then used to analyze water samples from Lake Vegoritis.

#### 2.5.1. Chromatographic Separation and MS/MS Identification of CPs

A sample of cyanobacterial mass from a bloom in Lake Kastoria, Greece (September 2014) containing all target CPs was used as a reference sample. Efficient chromatographic separation of target CPs was achieved with a reversed-phase C18 column (Atlantis T3, Waters), previously applied for MCs, NODs, CYN and ATX [33].

**Figure 4:**
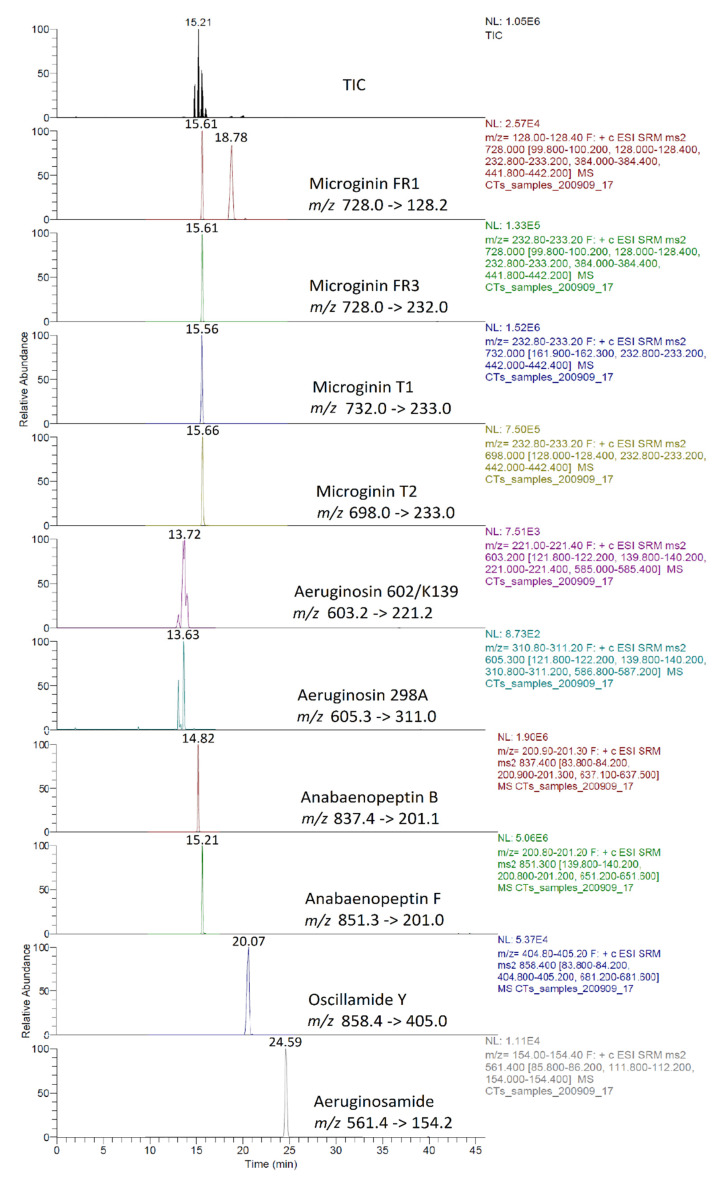
Example of TIC and MRM chromatograms of quantifier transitions for the intracellular fraction of CPs (sample taken on 7 September 2020, Site 1).

**Figure 5:**
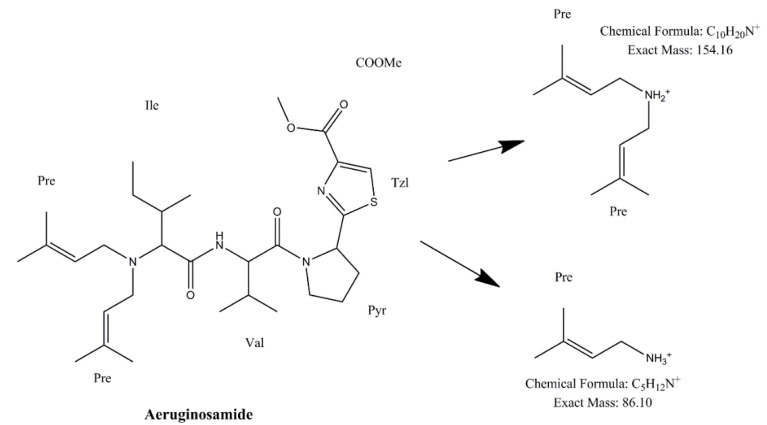
Structure of proposed fragment ions of AEG A.

Identification of CPs was performed using tandem mass spectrometry in a multiple reaction monitoring (MRM) mode (Table 2). The total ion chromatogram (TIC) and MRM chromatograms of the selected quantifier transitions obtained from cyanobacterial mass extract from Lake Vegoritis (7 September 2020, Site1) are presented in Figure 4. MGs presented a characteristic fragment ion, at *m/z* 128.2, attributed to a part of Ahda and, at *m/z* 162.1, to a part of chlorinated Ahda [44,45]. The most intense common ions of MGs, which share the amino acid sequence proline (Pro)-tyrosine (Tyr)-tyrosine (Tyr) at the C-terminus in their structure (i.e., MG FR3, MG T1 and MG T2), were at *m/z* 233.0 [Pro-Tyr-CO + H]^+^ and *m/z* 442.2 [Pro-Tyr-Tyr + H]^+^ [45,46]. AERs were characterized by *m/z* 140.0 and *m/z* 122.0, which are the Choi immonium ion and dehydrated Choi immonium ion, respectively [47]. Additionally, *m/z* 221.2 was attributed to Leucine (Leu)-Choi fragment (or Isoleucine (Ile)-Choi fragment), while *m/z* 311.0 was indicative of the presence of the arginine derivative—argininol in the structure of AERs [45,48]. APs were characterized by *m/z* 84, which corresponds to the lysine (Lys) immonium ion [49]. The fragment ion *m/z* 201.0 is characteristic of APs that contain arginine (Arg) as a side chain [49]. Fragment ions from the loss of the side chain amino acid with the CO linkage (i.e., *m/z* 637.3 for AP B and *m/z* 651.4 for AP F) or amino acid from the ring (i.e., *m/z* 681.4 for OSC Y [50]) were also considered. AEGs were characterized by *m/z* 112.0 and was annotated in a previous study as TzlCO in case of AEG A, since it is a common ion in the fragmentation spectra of some other AEGs [20]. The structure of fragment ions *m/z* 86.0 and *m/z* 154.2 was proposed in the frame of this study as [PreNH_3_]^+^ and [(Pre)_2_NH_2_]^+^, respectively (Figure 5). The fragmentation pathways of AEG A, involving the *m/z* 154.2 and 86.0 fragment ions based on in silico fragmentation (Mass Frontier 8.0, Thermo Scientific), are presented in Figure S3.

#### 2.5.2. Method Performance and Validation Results

The ability of the method for accurate quantification was assessed for AP B, for which an analytical reference standard was available. The response (quantification ion peak area) over the range 5–100 μg L^−1^ was linear (r^2^ ≥ 0.999). Precision, expressed as relative standard deviation (%RSD), was 8.6% under repeatability (*n* = 3) conditions and 15.9% under reproducibility (different days, *n* = 15) conditions. The limit of detection (LOD) of AP B was 0.001 μg/L and the LOQ was 0.003 μg/L. The LOD was estimated from measurements *(n* = 8) of standard solution (5 μg/L) using the formula: LOD = t (*n*−1, 0.95) × SD, where t (*n*−1, 0.95) was the t-test value for *n*—1 degrees of freedom at 95% confidence level, (1.895 for *n* = 8) and SD was the standard deviation of measurements. Limit of quantification (LOQ) was estimated as 3 × LOD.

Quantification of APs was carried out using the class equivalent approach with concentrations expressed as AP B equivalents, while for the rest CPs concentrations were expressed as MC-LR equivalents.

Recoveries of CPs (extracellular and intracellular fractions) were evaluated by analyzing spiked samples using CP-free water and cyanobacterial biomass as matrices and the reference sample from the Lake Kastoria bloom for spiking. Results are presented in Table 3. Recovery experiments were carried out in triplicate and mean recoveries in the extracellular fraction ranged from 77.0–129.2% for all target CPs, except for AEG A which was poorly recovered (17.1%) and AER 602/K139 that showed a recovery of 163.5%. Mean recoveries in the intracellular fraction were in the range of 73.4–98.3%, except for AEG A which had a low recovery (7.5%) (Table 3). In all recovery estimations, %RSD was <28.4%.

The validated analytical protocol can be used for detection and identification of target CTs and CPs of various chemical classes using a single analytical method. It could further serve as a basic template for analysis of cyanobacterial metabolites, expanding to more CTs and CPs in the future as they become commercially available as standards or included in mass spectral databases.

#### 2.5.3. Occurrence of CPs in Lake Vegoritis

Concentrations of extracellular and intracellular target CPs (MG FR1, MG FR3, MG T1, MG T2, AER 602/K139, AER 298A, AEG A, AP B, AP F, and OSC Y) during the monitoring period are presented in Tables S4 and S5, respectively.

**Figure 6:**
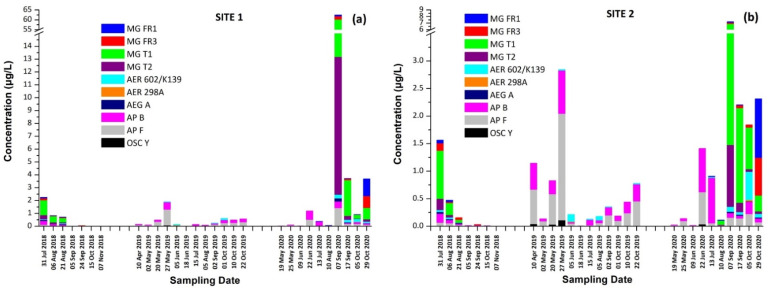
Occurrence of CPs detected in Lake Vegoritis at (**a**) Site 1 and (**b**) Site 2.

In 2018, MG FR1, MG FR3, MG T1, MG T2, AER 602/K139, and OSC Y were found only in intracellular fraction, with MG T1 to be the most abundant one, reaching 1.16 μg/L. AEG A, AP B, and AP F were found mostly in the intracellular fraction. AER 298A was not detected in any sample. During 2019, none of the samples analyzed were found to contain detectable amounts of MG FR1, MG FR3, MG T1, MG T2, and AEG A. AER 602/K139 and AER 298A were found only in the intracellular fraction. Target APs were all present in both extracellular and intracellular fractions, with the intracellular being at higher concentrations than the extracellular. In 2020, MG FR1, MG FR3, MG T1, MG T2, AER 298A, and AEG A were present only in intracellular form with MG T1 found to be the most abundant, reaching 47.0 μg/L. AER 602/K139, AP B, AP F, and OSC Y were found in both extracellular and intracellular form. AP F was the most abundant, up to 1.382 μg/L in the intracellular fraction, while AER 602/K139 was the most abundant (0.154 μg/L) in the extracellular fraction. The results present a similar trend in the CPs profile in both sampling sites (Figure 6).

**Figure 7:**
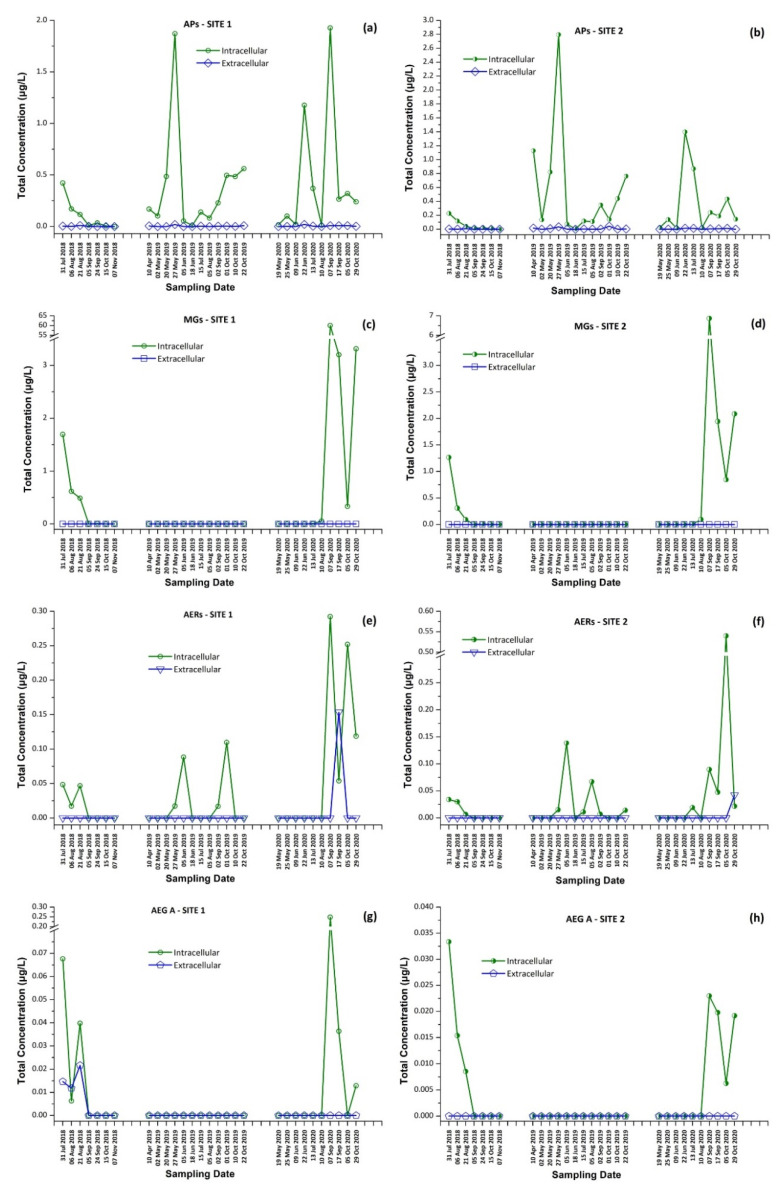
Intracellular and extracellular fractions of APs, MGs, AERs and AEG A per sampling date; APs at (**a**) Site 1 and (**b**) Site 2; MGs at (**c**) Site 1 and (**d**) Site 2; AERs at (**e**) Site 1 and (**f**) Site 2; and AEG A at (**g**) Site 1 and (**h**) Site 2.

These findings consist of the first report of the occurrence of a variety of CPs, in addition to MCs and CYN, in Lake Vegoritis (Figure S4), showing that all 10 target CPs were detected, with MGs and Aps found to be the most abundant classes of CPs compared to other classes (Figure 7). The highest concentration of total MGs (sum of intra- and extra-cellular), 60.0 μg/L, was measured on 7 September 2020 (Site 1), and consisted of MG FR1, MG FR3, MG T1, and MG T2, where MG T1 was the most abundant. The maximum of total APs (AP B, AP F, and OSC Y), 2.83 μg/L (Figure 2), was measured on 27 May 2019 (Site 2), with AP F presenting the highest concentration (Figure 6).

To date, there is only one report in the literature related to the presence of MGs in cyanobacterial bloom samples collected from Greek lakes other than Vegoritis, providing only qualitative data with no information on the presence of MGs in the water phase [32]. According to that study, the most frequently detected MGs were MG FR1 (70% of samples) followed by MG T1 (52%). In another study on the occurrence of APs in the freshwater bodies of Greece, the presence of AP B and AP A was reported, however, analysis was carried out using HPLC-UV without confirmation by mass spectrometry [51].

There is still lack of information concerning the occurrence of AERs and AEG A in Greek lakes. Furthermore, there is a gap in the knowledge related to the co-existence of CPs with CTs which may cause adverse health effects to humans and animals. In the frame of the present study, it was found that in water samples of Lake Vegoritis, AP B and AP F, which were detected in almost all samples (100% and 98%, respectively), co-existed with the frequently found MC-LR, CYN, and MC-RR (79%, 71%, and 50%, respectively), (Table 1). OSC Y was also present in 68% of the samples. The frequency of occurrence of the rest of the CPs ranged from 9% to 45%, while the detected CTs ranged from 5% to 24%, respectively.

Cyanobacteria possess a great metabolic potential and are able to co-produce several peptides from different classes [15]. MGs and AEG A exhibited a similar trend in Lake Vegoritis and their production could be attributed to *Microcystis* spp. [21,47,52], since both CP classes were detected in 2018 and 2020, but not in 2019, similarly to *Microcystis* spp. Low concentrations of AERs were determined in all sampling periods and their presence in Lake Vegoritis might be related to *Aphanizomenon* spp. and *Dolichospermum* spp. [53,54] as well as *Microcystis* spp. [18]. *Dolichospermum* spp. (*Anabaena* spp.), the dominant cyanobacterial species during all sampling periods, are possibly related to the high frequency of APs detection, especially AP B, which was present in all samples from Lake Vegoritis [55].

Our results are in agreement with other studies investigating the presence of CPs in inland water bodies. MCs, Aps, and AERs were the main cyanobacterial metabolites identified in biomass during a bloom episode in a dam for drinking water on Lake Occhito, near the town of Foggia in Southern Italy [56]. Similarly, MCs and APs were identified in the biomass from Siemianówka Dam Reservoir (northeast Poland) during a study from 2009 to 2012 [57]. In a more recent study of six eutrophic lakes in USA by Beversdorf et al., APs were detected in all lakes together with MGs at concentrations of the same order of magnitude found in Lake Vegoritis [22]. Similarly, both MCs and CPs (APs, MGs, and cyanopeptolins) were detected in surface and raw drinking waters from the eutrophic Lake Winnebago, Wisconsin [58]. In another study on the diversity and spatial distribution of MCs, NODs, Aps, and MGs in Green Bay, Lake Michigan, an important recreational resource, the presence of MCs (mainly MC-RR and -LR) and CPs (mainly APs and MGs) was reported with the mean of total MCs and APs as 1.28 and 0.20 μg/L, respectively [23].

The occurrence of cyanobacterial metabolites was also reported for lake water samples (mostly recreational) from Canada, in the frame of a collaborative citizen-science project. CTs were present in 75% of the samples, from ng/L up to μg/L, and AP A and AP B were in 38% of the samples at concentrations up to 10 μg/L [59]. High levels of APs (μg mg/L) were also detected in water samples from the Sau-Susqueda-El Pasteral reservoir system in Spain in the autumn of 2015, although MCs were <0.3 μg/L [60].

In most of the published studies, the intracellular (cell-bound) or total concentration (sum of extracellular and intracellular fractions) of CPs was reported. There is a general lack of knowledge on whether CPs are released from cyanobacterial cells into water or remain as cell-bound in the intracellular fraction [58]. In the frame of the current study, as intra- and extracellular fractions were quantified separately, it seems that the CPs studied are mostly intracellular, with the extracellular fraction concentrations being generally lower by an order of magnitude (Tables S4, S5 and Figure 7).

From Figure 2, it was also observed that the maximum concentrations of CPs occurred at the same time period with CTs, following the same trend. Taking into consideration the co-occurrence of CTs with CPs of different classes with unknown behavior, bioactivity and environmental levels in lake water, more research and monitoring programs are urgently needed for assessing possible threats to humans and the environment. This study was the first of this kind in Greece and in Lake Vegoritis, where both recreational and fishing activities take place.

## 3. Conclusions

This study reports, for the first time, the co-occurrence of CTs and CPs in Lake Vegoritis, situated in the north of Greece. Furthermore, the study describes the variant-specific changes of CT and CP profiles over a 3-year monitoring period as well as the basic water quality parameters of the lake and phytoplankton-cyanobacteria composition.

In order to realize the study, a previously validated LC-MS/MS analytical protocol for the simultaneous determination of multi-class cyanotoxins in water was further extended and validated to be applied for the detection of multiple CPs, i.e., MGs (MG FR1, MG FR3 MG T1, and MG T2), AERs (AER 602/K139 and AER 298A), AEG A and APs (AP B, AP F, and OSC Y). Samples of Lake Vegoritis were analyzed for CTs and CPs in both extracellular and intracellular fractions.

Using the above protocol, CTs were detected in two sampling sites of Lake Vegoritis, during 2018–2020, consisting of MC-LR, MC-RR, MC-YR, dmMC-LR, dmMC-RR, MC-HtyR, MC-HilR and CYN, with MC-LR (79%) being the most frequently detected, followed by CYN (71%). The concentrations of MCs and CYN were generally low (<1 μg/L) and they did not exceed the guideline values proposed for recreational water by WHO (24 and 6 μg/L for MC-LR and CYN, respectively). CPs belonging to the classes of APs, AERs, MGs, and AEG A were also detected, with AP B and AP F present in almost all water samples. The co-occurrence of two potent cyanotoxin classes (MCs and CYN) with multiple CPs in the lake’s water supports the need for future studies on the interactions between multiple cyanobacteria metabolites with regards to possible effects on human health.

## 4. Materials and Methods

### 4.1. Study Area Description and Sample Collection

**Figure 8:**
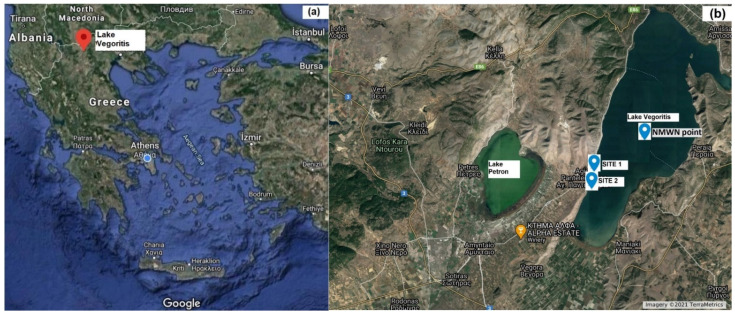
The (**a**) map and (**b**) sampling points of Lake Vegoritis.

Lake Vegoritis, one of the largest lakes in Greece, is located in Western Macedonia region in North-Western Greece and occupies the lowest area of the Ptolemaida basin (Figure 8a). It is considered as one of the most important water resources of Western Macedonia for its multiple uses and benefits for humans [34]. The investigative monitoring of the condition of Lake Vegoritis was designed and implemented from 2018 to 2020. In compliance with the WFD provisions [35], integrated samples (from the euphotic zone, 2.5× Secchi Depth) were taken from the pelagic zone of the lake (40°44′40.70′’N, 21°47′3.90′’E, National Monitoring Water Network sampling point, NMWN point), with a Nansen water sampler (Hydro-Bios, Germany). Physicochemical features were measured seasonally and each month during the growing season (May to October). For phytoplankton and chlorophyll α analysis, 2–4 samples were obtained during the growing season. Grab samples (1.5 L) for toxins analysis were collected in disposable plastic bottles from the surface layer of the lake (0–30 cm) at least monthly, from July to November, April to October, and May to October during 2018, 2019 and 2020, respectively. Sampling took place at two points: Site 1, 40°43′29.5′’N, 21°45′13.6′’E from the designated bathing area of the lake, and Site 2, 40°43′12.1′’N, 21°45′07.2′’E from the pier (Figure 8b). The samples were transported to the laboratory within 24 h of collection, in dark containers and at low temperature (≈4 °C).

### 4.2. Chemicals and Instrumentation

[D-Asp^3^]MC-LR, [D-Asp^3^]MC-RR, MC-WR, MC-HtyR, MC-HilR, MC-LY, MC-LW, MC-LF, and AP B standards were supplied by ENZO Life Science. MC-RR, MC-LR, MC-YR, MC-LA, and NOD standards were supplied by Sigma-Aldrich. CYN was purchased from Abraxis and (±) Anatoxin-a fumarate from TOCRIS Bioscience. All substances had a purity of >95%. Organic solvents i.e., acetonitrile (ACN) and methanol (MeOH) of HPLC grade (99.9%) and dichloromethane (DCM) of analytical reagent grade (99.9%), were supplied by Fisher Chemical. Formic acid (FA) was obtained from Riedel de Haen. Ultra-pure water (18.2 MΩ cm at 25 °C) was produced in the lab using a Temak TSDW10 water purification system (TEMAK, Athens, Greece).

Lake water samples were filtered through glass fiber filters with a 47 mm diameter and 0.7 μm pore size (Millipore, Cork, Ireland) and a glass vacuum filtration device. Solid-phase extraction (SPE) was carried out using a 12-port SPE vacuum manifold with large volume samplers connected through PTFE tubes (Supelco, Bellefonte, PA, USA) and a diaphragm vacuum pump (KNF Laboport, Freiburg, Germany). SPE cartridges used for cleanup and pre-concentration purposes were Supel-Select HLB (bed wt. 200 mg, volume 6 mL, Supelco, St. Louis, MO, USA) and Supelclean ENVI-Carb (bed wt. 250 mg, volume 3 mL, Supelco, St. Louis, MO, USA).

The analysis of target analytes was carried out using a TSQ Quantum Discovery Max triple-stage quadrupole mass spectrometer (Thermo Electron Corporation, San Jose, PA, USA), with an electrospray ionization (ESI) source coupled to a Finnigan Surveyor LC system, equipped with a Surveyor AS autosampler (Thermo Electron Corporation, San Jose, PA, USA). Xcalibur software 2.0 was used to control the MS parameters for data acquisition and data analysis. The chromatographic column used was an Atlantis T3 (2.1 mm × 100 mm, 3 μm, Waters, Wexford, Ireland).

### 4.3. Physico-Chemical Parameters

Ion chromatography (IC) was applied for separation, analysis, and quantification of both anions (F^−^, Cl^−^, NO_2_^−^, Br^−^, NO_3_^−^, PO_4_^3−^, and SO_4_^2−^) and cations (Na^+^, K^+^, Mg^2+^, and Ca^2+^) in water samples. Subsamples were filtered through 0.45 μm glass fiber filters within 48 h of sampling and kept at 2–6 °C for up to 4 days. For cation analysis, samples were acidified to a pH of 3 ± 0.5 with nitric acid. Dissolved anions and cations were determined using a Dionex Aquion IC System (Thermo Fisher Scientific, Waltham, MA, USA) according to EN ISO 10,304 and 14,911 guidelines, respectively [61,62].

Determination of total phosphorus (TP) was carried out in unfiltered water subsamples (about 250 mL, stored at −18 °C for up to six months), using the ascorbic acid method following persulfate digestion [63]. Orthophosphates were determined based on spectrophotometry (Hitachi U-5100 UV/VIS, Hitachi, Tokyo, Japan).

For determination of total suspended solids (TSS), a measured volume of a water subsample (about 500 mL) was filtered through a pre-weighed glass microfiber filter (Whatman Grade 934-AH^®^ RTU circles). The filter was heated at 103–105 °C for an hour and then weighed. The TSS were calculated as the mass increase divided by the water volume filtered [63]. Analysis was conducted within seven days from sampling and the subsamples were kept at 4 °C in the dark.

### 4.4. Chlorophyll α Analysis

Subsamples of 1 L were filtered through Whatman GF/F glass fiber filters within 48 h of sampling. Chlorophyll α was measured using 90% acetone and application of the trichromatic equation [63,64]. Absorbance was measured using a Cary 60 UV-Vis spectrophotometer (Agilent Technologies, Santa Clara, CA, USA).

### 4.5. Microscopic Analysis

For phytoplankton analysis, subsamples (about 500 mL) were transferred to plastic bottles and preserved with acid Lugol’s solution [65]. Additional samples were obtained by vertical plankton net hauls (20 μm mesh, Hydro-Bios, Altenholz, Germany) through the euphotic zone, and preserved with formaldehyde. Phytoplankton identification and enumeration were based on the Utermöhl settling technique [66] as described in EN 15,204 guidelines [65]. The analysis was carried out using sedimentation chambers (Hydro-Bios) and an inverted microscope Zeiss AxioObserver.A1 equipped with an AxioCam (Carl Zeiss, Oberkochen, Germany). The phytoplankton biomass was estimated following EN 16,695 guidelines [67].

### 4.6. Analysis of CTs and CPs

#### 4.6.1. Sample Preparation

Analysis of CTs (CYN, ATX, NOD, dmMC-RR, MC-RR, MC-YR, MC-HtyR, dmMC-LR, MC-LR, MC-HilR, MC-WR, MC-LA, MC-LY, MC-LW, and MC-LF) and CPs (MG FR1, MG FR3, MG T1, MG T2, AER 602/K139, AER 298A, AEG A, AP B, AP F, and OSC Y) in water samples was carried out by liquid chromatography coupled with tandem mass spectrometry (LC-MS/MS). For the determination of intra- and extracellular CTs and CPs, water samples were first filtered through GF/F filters and then the filters and filtered water were analyzed. Intracellular CTs and CPs were extracted from the filters’ biomass by an extraction mixture containing 75% MeOH:25% H_2_O. After evaporation of the extract and reconstitution with MeOH: H_2_O (5:95 *v/v*), the final solution was injected into the LC-MS/MS for analysis [31]. Filtered water samples were pre-treated using the dual cartridge (HLB and Envi-Carb) SPE process [33]. Briefly, water samples, after adjustment to pH 11, were passed through a dual cartridge assembly of HLB and ENVI-Carb. Recovery of extracellular CTs and CPs was achieved by reversing the cartridges and eluting with a mixture of 10 mL DCM:MeOH (40:60, *v/v*), containing 0.5% FA. The extract was dried and the residue was re-dissolved with 400 μL MeOH: H_2_O (5:95, *v/v*) prior to LC-MS/MS analysis.

#### 4.6.2. LC-MS/MS Analysis

A gradient elution program was applied for chromatographic separation with solvents (A) ACN and (B) water, both containing 0.5% FA. The gradient started at 5% A (held for 3 min), which increased to 20% A in 1 min (held for 2 min), further to 35% A in 1 min (held for 7 min), 70% A in 14 min, and finally 90% in 1 min (held for 3 min). An equilibration time of 10 min was kept after each sample run. The flow rate was set at 0.2 mL/min with 20 μL injection volume and the column temperature was set at 30 °C.

Electrospray ionization (ESI) in positive mode was used and the three most intense and characteristic precursor–product ion transitions were selected for detection and identification of each compound in MRM mode. LC-MS/MS detection parameters for the target CTs was set according to Zervou et al. [33]. Parameters for the CPs detection are given in Table 2. The selection of MRM transitions was based on fragmentation spectra from previous studies [46,49,50,57,68] or in the frame of this study. In all cases, single protonated [M+H]^+^ ions were set as the precursor ions. The most intense product ion was chosen to be the quantifier ion, while quantification was performed using external standards at concentrations of 5, 20, and 100 μg/L. For APs, since only AP B was commercially available as a standard, quantification was carried out using the class equivalent approach with AP F and OSC Y concentrations expressed as AP B equivalents. All other CPs for which no class equivalent standard was available, were expressed as MC-LR equivalents [69].

#### 4.6.3. Validation of Methods for the Determination of CPs

The analytical work flow used for the target CTs [31] was also validated for its performance for CPs. Recovery studies were carried out by spiking samples with an extract of cyanobacterial mass (Lake Kastoria, September 2014) containing all target CPs. The TIC and MRM chromatograms of the selected quantification ions obtained from cyanobacterial mass extract from Lake Kastoria (September 2014) are shown in Figure S5.

To obtain the extract of cyanobacterial mass used for spiking, 10 mg of lyophilized biomass (Lake Kastoria, September 2014) was extracted two times with 1.5 mL of 75% MeOH: 25% H_2_O and a third time with 1.5 mL *n*-butanol. Each time the mixture was vortexed, sonicated for 15 min in a sonication bath (Bandelin Sonorex Super RK106) and then centrifuged at 4000 rpm for 10 min at room temperature (DuPont RMC-14 Refrigerated Micro-Centrifuge, Sorvall Instruments, Newtown, CT, USA) and the supernatant was separated from the pellet. All supernatants were pooled together. One milliliter of the extract was evaporated to dryness, the residue was re-dissolved in 400 μL of MeOH: H_2_O (5:95 *v/v*) and analyzed by LC-MS/MS. The rest of the extract was used for spiking CP-free biomass (retained on filter after passing 200 mL of lake water) and filtered water samples (400 mL) to carry out recovery experiments. Additionally, recovery experiments were carried out by spiking filtered CP-free water samples (400 mL) with AP B at the concentration level of 100 ng/L.

## Figures and Tables

**Table 1:** Percentage of samples where cyanotoxins (CTs) and cyanopeptides (CPs) were detected during the monitoring period (2018–2020).

CTs																
	CYN		dm MC-RR		MC-RR		MC-YR		MC-HtyR		dm MC-LR		MC-LR		MC-HilR	
% Presence	71		12		50		24		7		17		79		5	
**CPs**																
	AP B	AP F		Osc Y		MG FR1		MG FR3	MG T1	MG T2		AER 602/K139		AER 298A		AEG A
% Presence	100	98		68		27		27	29	29		45		9		23

**Table 2:** LC–MS/MS detection parameters of CPs.

Cyanopeptide	t_R_ (min)	Precursor Ion	Product Ions	Collision Energy (eV)	Product Ion Assignment	Ref.
MG FR1	18.9	728.0 [M+H]^+^	100.0	40	MeLeu immonium ion	[46]
			128.2 ^Q^	40	Ahda fragment (C_8_H_18_N)	[45]
			384.2	40	[M + H-Tyr-Tyr]^+^	[46]
MG FR3	15.6	728.0 [Μ+H]^+^	128.2	40	Ahda fragment (C_8_H_18_N)	[45]
			233.0 ^Q^	40	[Pro-Tyr-CO + H]^+^	[45]
			442.0	40	[Pro-Tyr-Tyr + H]^+^	[46]
MG T1	15.5	732.0 [Μ+H]^+^	162.1	40	Cl-Ahda fragment (C_8_H_17_NCl)	[44]
			233.0 ^Q^	40	[Pro-Tyr-CO + H]^+^	[45]
			442.2	40	[Pro-Tyr-Tyr + H]^+^	[46]
MG T2	15.7	698.0 [M+H]^+^	128.2	40	Ahda fragment (C_8_H_18_N)	[45]
			233.0 ^Q^	40	[Pro-Tyr-CO + H]^+^	[45]
			442.2	40	[Pro-Tyr-Tyr + H]^+^	[46]
AER 602/Κ139	13.8	603.2 [Μ+H]^+^	122.0	40	[Choi immonium-H_2_O]^+^	[47]
			140.0	40	Choi immonium ion	[47]
			221.2 ^Q^	40	Leu-Choi fragment	[45]
AER 298A	13.6	605.3 [Μ+H]^+^	122.0	40	[Choi immonium-H_2_O]^+^	[47]
			140.0	40	Choi immonium ion	[47]
			311.0 ^Q^	40	[Choi-Argininol-NH_2_ + H]^+^	[48]
AEG A	24.6	561.4 [Μ+H]^+^	86.0	40	[PreNH_3_]^+^	This study
			112.0	40	TzlCO	[20]
			154.2 ^Q^	40	[(Pre)_2_NH_2_]^+^	This study
AP B	14.8	837.4 [Μ+H]^+^	84.0	40	Lys immonium ion	[49]
			201.1 ^Q^	40	CO-Arg (side chain)	[49]
			637.3	40	[Lys-Phe-MeAla-HTyr-Val + 2H]^+^	[49]
AP F	15.2	851.3 [Μ+H]^+^	84.0	40	Lys immonium ion	[49]
			201.0 ^Q^	40	CO-Arg (side chain)	[49]
			651.4	40	[Lys-Phe-MeAla-HTyr-Ile + 2H]^+^	[49]
OSC Y	19.9	858.4 [Μ+H]^+^	84.0	40	Lys immonium ion	[50]
			405.0 ^Q^	40	[M + H-Tyr-(Htyr-Ile)]^+^	[50]
			681.4	40	[M + H-Htyr]^+^	[50]

**^Q^** quantifier ion.

**Table 3:** Recoveries of target CPs from water (extracellular and intracellular).

	MG FR1	MG FR3	MG T1	MG T2	AER 602/K139	AER 298A	AEG A	AP B	AP F	Osc Y
Extracellular Recovery (%RSD, *n* = 3)	103.7% (9.5)	79.7% (9.2)	86.5% (6.7)	77.0% (6.3)	163.5% (7.4)	129.2% (8.8)	17.1% (25.2)	102.7 (8.6)	108.6% (6.5)	95.8% (6.5)
Intracellular Recovery (%RSD, *n* = 3)	74.0% (16.9)	75.5% (2.2)	75.1% (9.9)	75.6% (13.8)	88.8% (5.6)	98.3% (13.7)	7.5% (28.4)	87.2% (3.3)	96.5% (9.1)	73.4% (10.0)

## Data Availability

Data is contained within the article or Supplementary Material.

## Supplementary Materials

The following are available online at https://www.mdpi.com/article/10.3390/toxins13060394/s1, Figure S1: structures of several classes of cyanotoxins. For the classes of *Microcystins* and *Nodularins*, R_x_ stands for variable L-amino acids, Figure S2: structures of several classes of cyanopeptides. For the classes of *Anabaenopeptins* and *Microginins*, A_x_ stands for variable L-amino acids, Figure S3: fragmentation pathways of AEG A involving the *m/z* 154.2 and 86.0 fragment ions. Fragmentation pathways predicted by Mass Frontier 8.0 software, Figure S4: heat maps showing all CP and CT concentrations detected in Lake Vegoritis throughout the two sampling points (Site 1 and Site 2), Figure S5: TIC and MRM chromatograms of quantifier ions for the cyanopeptides obtained from the biomass extract of Lake Kastoria (sampling September 2014), and Table S1: physico-chemical parameters, total phytoplankton biomass and cyanobacteria biomass concentrations measured during the study period. Table S2: concentrations of extracellular CTs from Lake Vegoritis (μg/L), Table S3: concentrations of intracellular CTs from Lake Vegoritis (μg/L), Table S4: concentrations of extracellular CPs from Lake Vegoritis (μg/L), and Table S5: concentrations of intracellular CPs from Lake Vegoritis (μg/L).
