# Supplementary material for: Cyanobacterial Toxins and Peptides in Lake Vegoritis, Greece
Source: Toxins (Basel). 2021 Jun 1;13(6):394. doi: 10.3390/toxins13060394 (PMC8230288; doi:10.3390/toxins13060394)
Supplement: Supplementary file 1 [file toxins-13-00394-s001.zip › toxins-1221232 Supplementary.pdf]

Supplementary Materials: Cyanobacterial Toxins and Peptides in Lake Vegoritis, Greece

Sevasti-Kiriaki Zervou, Kimon Moschandreou, Aikaterina Paraskevopoulou, Christophoros Christophoridis, Elpida Grigoriadou, Triantafyllos Kaloudis, Theodoros M. Triantis, Vasiliki Tsiaoussi and Anastasia Hiskia

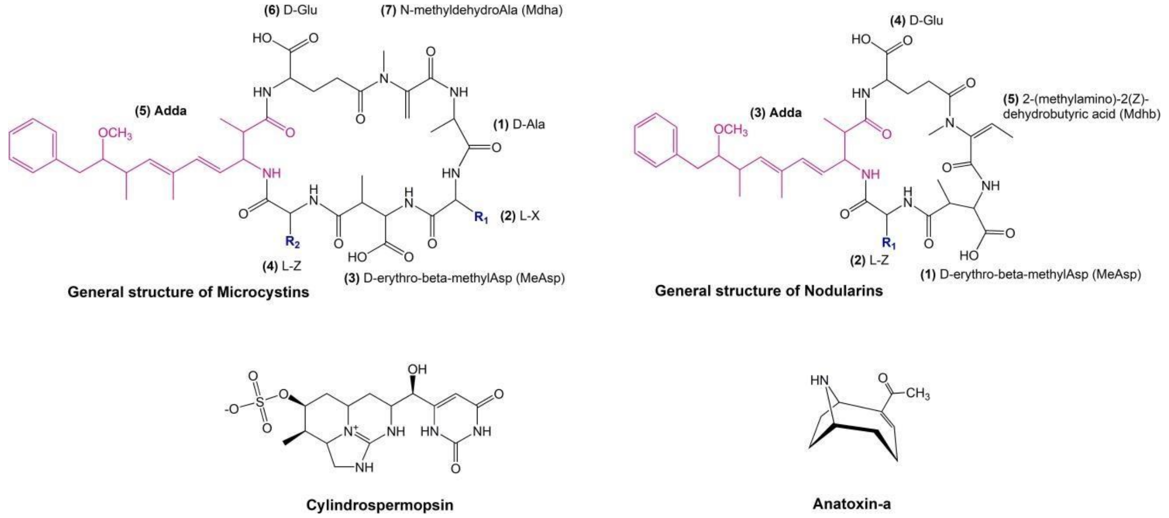

Figure S1. Structures of several classes of cyanotoxins. For the classes of Microcystins and Nodularins, R_x stands for variable L-amino acids.

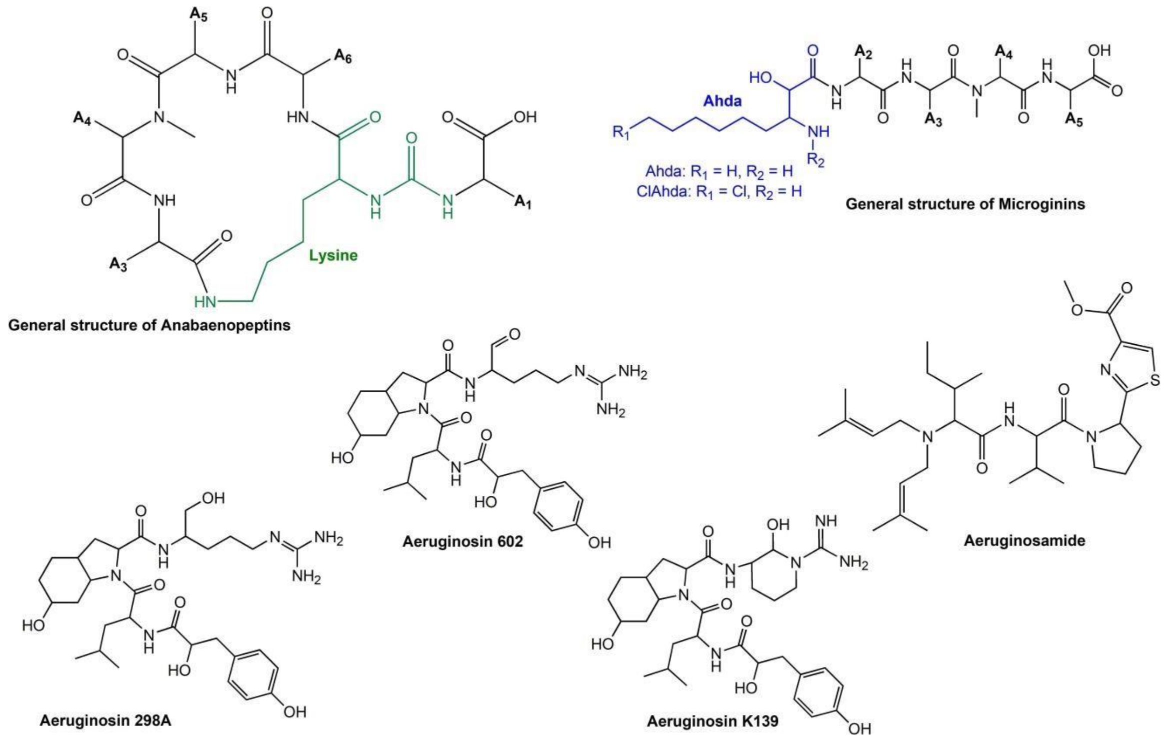

Figure S2. Structures of several classes of cyanopeptides. For the classes of Anabaenopeptins and Microginins, A_x stands for variable L-amino acids.

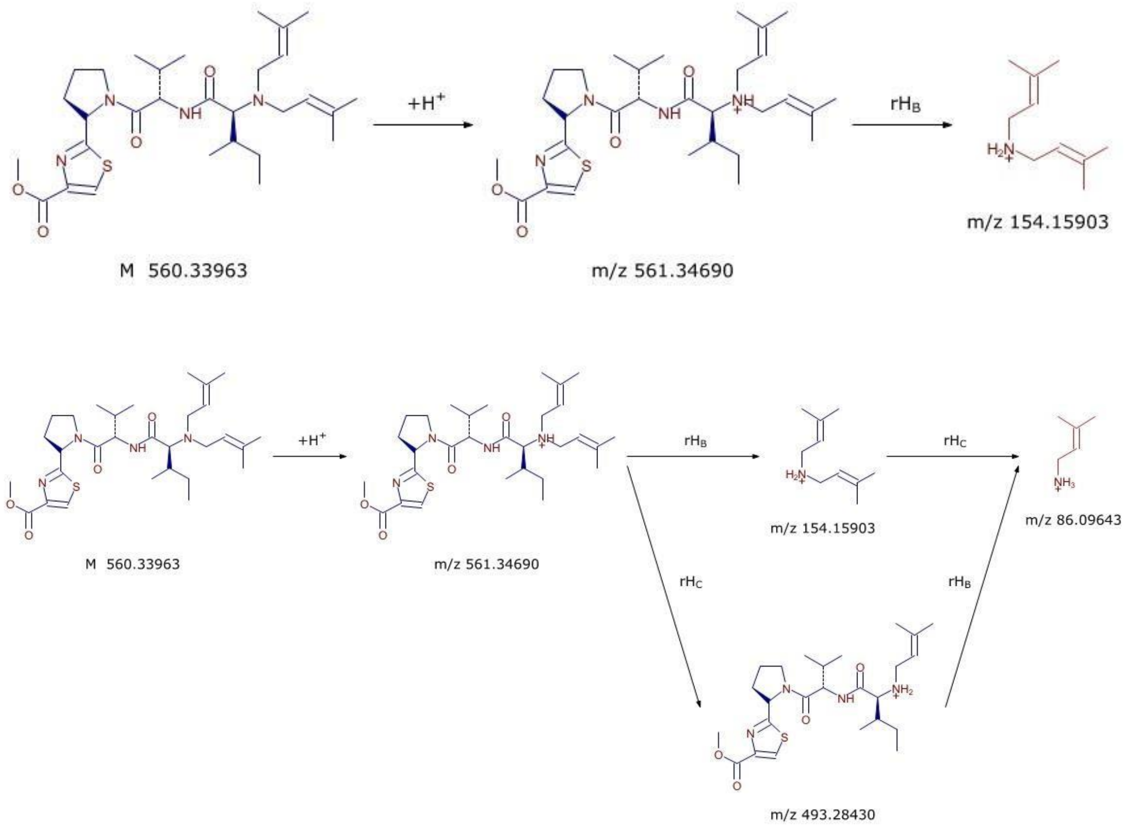

Figure S3. Fragmentation pathways of AEG A involving the *m/z* 154.2 and 86.0 fragment ions. Fragmentation pathways predicted by Mass Frontier 8.0 software.

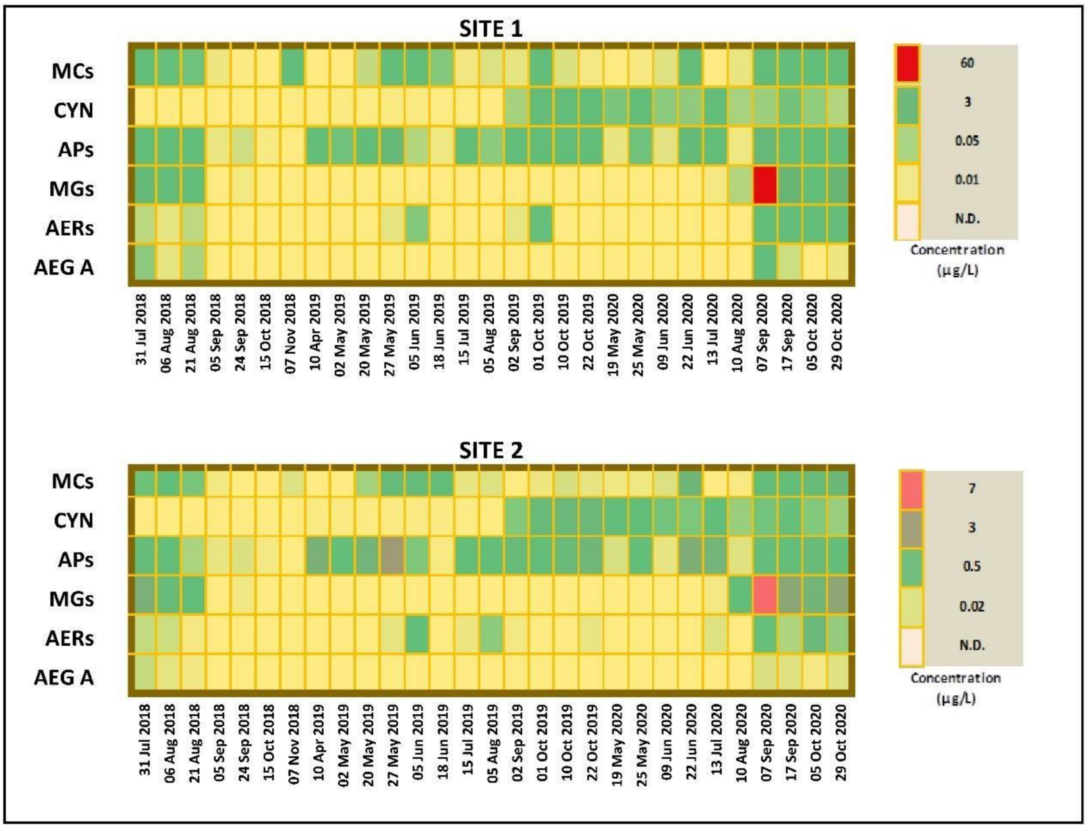

Figure S4. Heat maps showing all CP and CT concentrations detected in Lake Vegoritis throughout the two sampling points (Site 1 and Site 2).

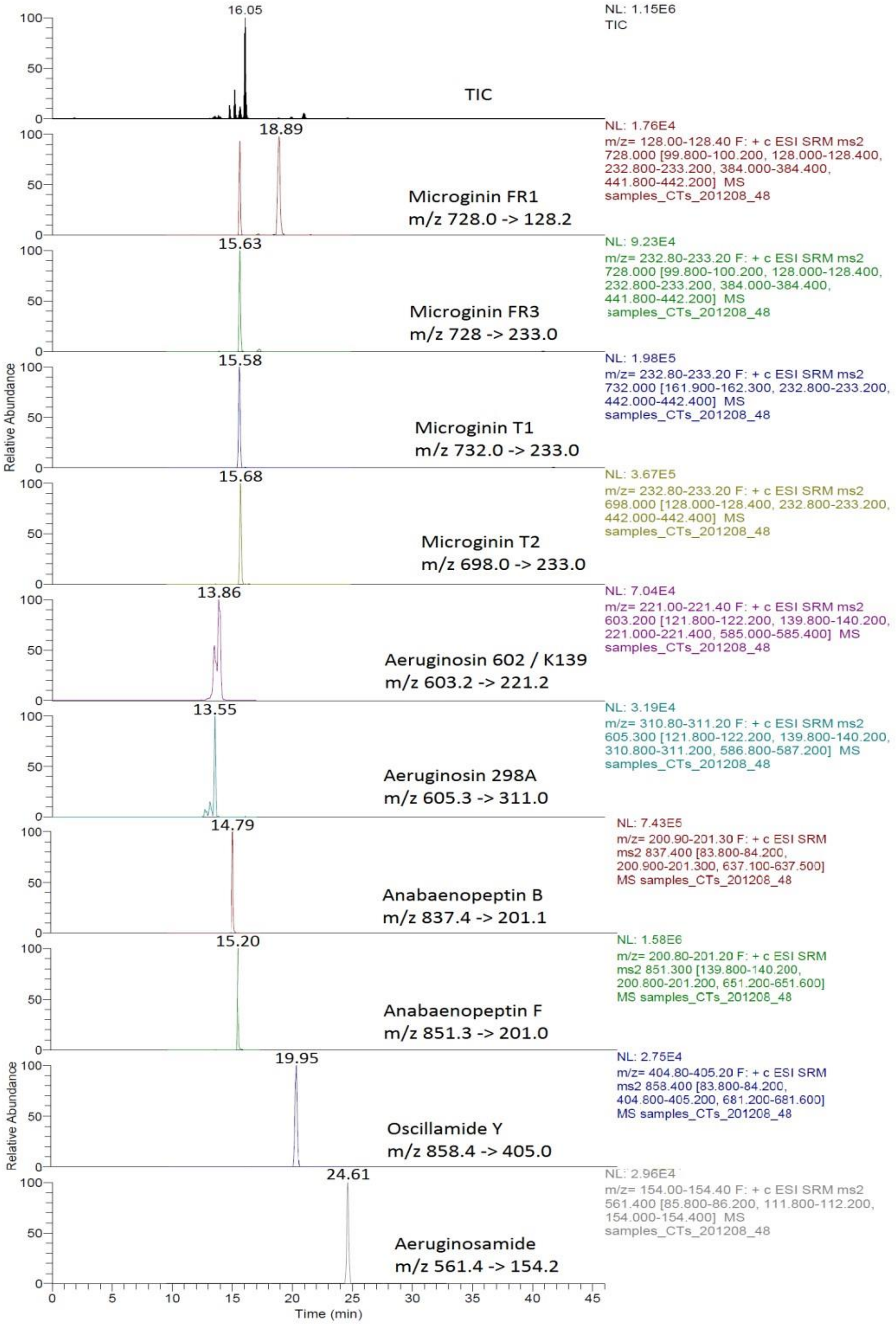

Figure S5. TIC and MRM chromatograms of quantifier ions for the cyanopeptides obtained from the biomass extract of Lake Kastoria (sampling September 2014).

Table S1. Physico-chemical parameters, total phytoplankton biomass and cyanobacteria biomass concentrations measured during the study period.

NMWN sampling point																		
Sampling Date	Chl-α (μg/L)	Total Biomass (mg/L)	Cyanobacteria Biomass (mg/L)	TP (mg/L)	Alkalinity (meq/L)	TSS (mg/L)	Transparency (Secchi disc depth in cm)	F^- (mg/L)	Cl^- (mg/L)	NO2- (mg/L)	Br^- (mg/L)	NO3- (mg/L)	PO43- (mg/L)	SO42- (mg/L)	Na^+ (mg/L)	K^+ (mg/L)	Mg^{2+} (mg/L)	Ca^{2+} (mg/L)
5-Feb-18				0.060	4.75	1.07	650.00	<LOQ	36	<LOQ	<LOQ	0.93	<LOQ	200	35	5.4	45	38
25-Apr-18				0.050	4.91	1.41	600.00	<LOQ	36	<LOQ	<LOQ	0.53	<LOQ	89	36	5.8	45	44
18-Jun-18	15.85	2.299	1.180	0.043	4.04	8.46	30.00	<LOQ	35	<LOQ	<LOQ	<LOQ	<LOQ	87	36	6.0	45	26
4-Jul-18	8.01			0.040	4.05	2.86	240.00	<LOQ	35	<LOQ	<LOQ	0.37	<LOQ	160	37	6.2	46	32
12-Jul-18	7.92			0.036	4.11	2.96	300.00	<LOQ	35	<LOQ	<LOQ	0.53	<LOQ	93	37	5.9	46	32
17-Jul-18	7.18	1.428	0.793	0.040	4.09	2.63	280.00	<LOQ	34	<LOQ	<LOQ	<LOQ	<LOQ	99	34	5.7	43	29
6-Aug-18	4.70	1.318	1.082	<LOQ	4.29	2.41	420.00	<LOQ	38	<LOQ	<LOQ	1.04	<LOQ	100	37	5.8	45	33
5-Sep-18	3.18			<LOQ	4.40		500.00											
24-Sep-18				<LOQ			720.00											
30-Oct-18				0.022	4.48	1.99	320.00											
23-Jan-19				0.050	4.62	0.62	480.00	<LOQ	33		<LOQ			92	36	6.3	46	39
4-Feb-19				0.037		2.54	160.00	<LOQ	34	<LOQ	<LOQ	<LOQ	<LOQ	144	37	6.1	46	40
20-May-19	4.64	0.643	0.219	0.044	4.68	1.99	200.00	<LOQ	32	<LOQ	<LOQ	<LOQ	<LOQ	89	36	6.1	46	40
19-Jun-19	6.32	1.433	0.405	0.031	4.83	3.75	240.00	<LOQ	33	<LOQ	<LOQ	<LOQ	<LOQ	96	36	5.9	46	38
17-Jul-19	7.69	2.496	1.033	0.028	4.32	2.44	350.00	<LOQ	35	<LOQ	<LOQ	<LOQ	<LOQ	99	37	6.1	46	33
10-Sep-19	19.38	6.633	5.430	0.034	4.18	2.16	200.00	<LOQ	36	<LOQ	<LOQ	<LOQ	<LOQ	99	38	6.2	47	29
21-Oct-19				<LOQ	4.50	2.03	260.00	<LOQ	34		<LOQ			93	37	6.0	47	35
29-Jan-20						1.00	480.00	<LOQ	34	<LOQ	<LOQ	0.71	<LOQ	97	37	6.1	47	37
21-May-20	4.24			0.025		2.31	340.00	<LOQ	40	<LOQ	<LOQ	<LOQ	<LOQ	94	35	5.9	44	36
9-Jun-20	5.31	1.272	0.131	0.038		1.43	280.00	<LOQ	34	<LOQ	<LOQ	<LOQ	<LOQ	142	37	6.0	47	38
7-Jul-20	5.35	1.908	1.494	0.016		1.45	440.00	<LOQ	58	<LOQ	<LOQ	<LOQ	<LOQ	100	37	6.1	47	37
24-Aug-20		3.401	1.773	0.018		1.48	300.00	<LOQ	35	<LOQ	<LOQ	<LOQ	<LOQ	97	37	6.0	47	31
22-Sep-20	31.77	2.698	1.420	0.029		5.67	220.00	<LOQ	36	<LOQ	<LOQ	0.38	<LOQ	108	36	5.9	45	25
19-Oct-20				0.021		2.10	300.00	<LOQ	37	<LOQ	<LOQ	<LOQ	<LOQ	103	39	6.0	48	29
Bathing area sampling point (Site 1)																		
Sampling Date	Chl-α (μg/L)	Total Biomass (mg/L)	Cyanobacteria Biomass (mg/L)	TP (mg/L)	Alkalinity (meq/L)	TSS (mg/L)	Transparency (Secchi disc depth in cm)	F^- (mg/L)	Cl^- (mg/L)	NO_2- (mg/L)	Br- (mg/L)	NO3- (mg/L)	PO43- (mg/L)	SO42- (mg/L)	Na^+ (mg/L)	K^+ (mg/L)	Mg^{2+} (mg/L)	Ca^{2+} (mg/L)
4-Jul-18	6.53		1.358	0.033	4.04	1.71	240.00	<LOQ	35	<LOQ	<LOQ	0.44	<LOQ	98	38	8.4	46	32
12-Jul-18	5.83		0.850	0.047	4.13	3.23	290.00	<LOQ	35	<LOQ	<LOQ	0.81	<LOQ	121	38	6.2	46	34
17-Jul-18	6.66		1.432	0.033	4.31	2.56	290.00	<LOQ	34	<LOQ	<LOQ	<LOQ	<LOQ	84	34	5.4	43	30
6-Aug-18	3.29		0.359	<LOQ	4.26			<LOQ	38	<LOQ	<LOQ	0.25	<LOQ	98	36	5.5	45	35
5-Sep-18	3.03		0.010	<LOQ	4.30	0.61	500.00											
24-Sep-18	4.73		0.030	<LOQ	4.41	0.65	580.00											
15-Oct-18	5.32			<LOQ	4.52	0.59	460.00											

Table S2. Concentrations of Extracellular CTs from Lake Vegoritis (μg/L).

ID	SAMPLE DETAILS	CYN	ATX	dmMC-RR	MC-RR	NOD	MC-YR	MC-HtyR	dmMC-LR	MC-LR	MC-HilR	MC-WR	MC-LA	MC-LY	MC-LW	MC-LF
**1**	31/7/2018 – Site 1	ND[1]	ND	ND	**<LOQ**	ND	ND	ND	ND	ND	ND	ND	ND	ND	ND	ND
**2**	31/7/2018 – Site 2	ND	ND	ND	**<LOQ**	ND	ND	ND	ND	ND	ND	ND	ND	ND	ND	ND
**3**	6/8/2018 – Site 1	ND	ND	ND	**0.023**	ND	ND	ND	ND	**0.021**	ND	ND	ND	ND	ND	ND
**4**	6/8/2018 – Site 2	ND	ND	ND	**0.017**	ND	**<LOQ**	ND	ND	**0.013**	ND	ND	ND	ND	ND	ND
**5**	21/8/2018 – Site 1	ND	ND	ND	**0.020**	ND	**0.014**	ND	ND	**0.028**	ND	ND	ND	ND	ND	ND
**6**	21/8/2018 – Site 2	ND	ND	ND	**0.023**	ND	**<LOQ**	ND	ND	**0.029**	ND	ND	ND	ND	ND	ND
**7**	5/9/2018 – Site 1	ND	ND	ND	**<LOQ**	ND	ND	ND	ND	**<LOQ**	ND	ND	ND	ND	ND	ND
**8**	5/9/2018 – Site 2	ND	ND	ND	**<LOQ**	ND	ND	ND	ND	ND	ND	ND	ND	ND	ND	ND
**9**	24/9/2018 – Site 1	ND	ND	ND	**<LOQ**	ND	ND	ND	ND	ND	ND	ND	ND	ND	ND	ND
**10**	24/9/2018 – Site 2	ND	ND	ND	**<LOQ**	ND	ND	ND	ND	ND	ND	ND	ND	ND	ND	ND
**11**	15/10/2018 – Site 1	ND	ND	ND	ND	ND	ND	ND	ND	ND	ND	ND	ND	ND	ND	ND
**12**	15/10/2018 – Site 2	ND	ND	ND	ND	ND	ND	ND	ND	ND	ND	ND	ND	ND	ND	ND
**13**	7/11/2018 – Site 1	ND	ND	ND	**0.009**	ND	ND	ND	ND	**0.029**	ND	ND	ND	ND	ND	ND
**14**	7/11/2018 – Site 2	ND	ND	ND	ND	ND	ND	ND	ND	ND	ND	ND	ND	ND	ND	ND
**15**	10/4/2019 – Site 1	ND	ND	ND	ND	ND	ND	ND	ND	ND	ND	ND	ND	ND	ND	ND
**16**	10/4/2019 – Site 2	**0.004**	ND	ND	ND	ND	ND	ND	ND	ND	ND	ND	ND	ND	ND	ND
**17**	2/5/2019 – Site 1	**<LOQ**	ND	ND	ND	ND	ND	ND	ND	ND	ND	ND	ND	ND	ND	ND
**18**	2/5/2019 – Site 2	**<LOQ**	ND	ND	ND	ND	ND	ND	ND	ND	ND	ND	ND	ND	ND	ND
**19**	20/5/2019 – Site 1	**<LOQ**	ND	ND	ND	ND	ND	ND	ND	**<LOQ**	ND	ND	ND	ND	ND	ND
**20**	20/5/2019 – Site 2	**<LOQ**	ND	ND	ND	ND	ND	ND	ND	ND	ND	ND	ND	ND	ND	ND
**21**	27/5/2019 – Site 1	**<LOQ**	ND	ND	ND	ND	ND	ND	ND	**0.071**	ND	ND	ND	ND	ND	ND
**22**	27/5/2019 – Site 2	**<LOQ**	ND	ND	ND	ND	ND	ND	ND	**0.086**	ND	ND	ND	ND	ND	ND
**23**	5/6/2019 – Site 1	**<LOQ**	ND	ND	ND	ND	ND	ND	**0.073**	**0.188**	ND	ND	ND	ND	ND	ND
**24**	5/6/2019 – Site 2	**0.004**	ND	ND	ND	ND	ND	ND	**0.080**	**0.233**	ND	ND	ND	ND	ND	ND
**25**	18/6/2019 – Site 1	**<LOQ**	ND	ND	ND	ND	ND	ND	ND	**0.089**	ND	ND	ND	ND	ND	ND
**26**	18/6/2019 – Site 2	**<LOQ**	ND	ND	ND	ND	ND	ND	ND	**0.092**	ND	ND	ND	ND	ND	ND
**27**	15/7/2019 – Site 1	**<LOQ**	ND	ND	ND	ND	ND	ND	ND	ND	ND	ND	ND	ND	ND	ND

**28**	15/7/2019 – Site 2	**<LOQ**	ND	ND	ND	ND	ND	ND	ND	**0.013**	ND	ND	ND	ND	ND	ND
**29**	5/8/2019 – Site 1	ND	ND	ND	ND	ND	ND	ND	ND	**0.015**	ND	ND	ND	ND	ND	ND
**30**	5/8/2019 – Site 2	ND	ND	ND	ND	ND	ND	ND	ND	**<LOQ**	ND	ND	ND	ND	ND	ND
**31**	2/9/2019 – Site 1	**0.032**	ND	ND	ND	ND	ND	ND	ND	ND	ND	ND	ND	ND	ND	ND
**32**	2/9/2019 – Site 2	**0.026**	ND	ND	ND	ND	ND	ND	ND	ND	ND	ND	ND	ND	ND	ND
**33**	1/10/2019 – Site 1	**0.042**	ND	ND	ND	ND	ND	ND	ND	ND	ND	ND	ND	ND	ND	ND
**34**	1/10/2019 – Site 2	**0.089**	ND	ND	ND	ND	ND	ND	ND	ND	ND	ND	ND	ND	ND	ND
**35**	10/10/2019 – Site 1	**0.014**	ND	ND	ND	ND	ND	ND	ND	ND	ND	ND	ND	ND	ND	ND
**36**	10/10/2019 – Site 2	**0.019**	ND	ND	ND	ND	ND	ND	ND	ND	ND	ND	ND	ND	ND	ND
**37**	22/10/2019 – Site 1	**0.114**	ND	ND	ND	ND	ND	ND	ND	ND	ND	ND	ND	ND	ND	ND
**38**	22/10/2019 – Site 2	**0.027**	ND	ND	ND	ND	ND	ND	ND	ND	ND	ND	ND	ND	ND	ND
**39**	19/5/2020 – Site 1	**0.095**	ND	ND	ND	ND	ND	ND	ND	ND	ND	ND	ND	ND	ND	ND
**40**	19/5/2020 – Site 2	**0.104**	ND	ND	ND	ND	ND	ND	ND	ND	ND	ND	ND	ND	ND	ND
**41**	25/5/2020 – Site 1	**0.112**	ND	ND	ND	ND	ND	ND	ND	**<LOQ**	ND	ND	ND	ND	ND	ND
**42**	25/5/2020 – Site 2	**0.107**	ND	ND	ND	ND	ND	ND	ND	**<LOQ**	ND	ND	ND	ND	ND	ND
**43**	9/6/2020 – Site 1	**0.081**	ND	ND	ND	ND	ND	ND	ND	**0.023**	ND	ND	ND	ND	ND	ND
**44**	9/6/2020 – Site 2	**0.081**	ND	ND	ND	ND	ND	ND	ND	**0.021**	ND	ND	ND	ND	ND	ND
**45**	22/6/2020 – Site 1	**0.080**	ND	ND	ND	ND	ND	**0.069**	**0.036**	**0.285**	**0.033**	ND	ND	ND	ND	ND
**46**	22/6/2020 – Site 2	**0.077**	ND	ND	ND	ND	**0.020**	**0.069**	**0.035**	**0.315**	**0.029**	ND	ND	ND	ND	ND
**47**	13/7/2020 – Site 1	**0.128**	ND	ND	ND	ND	ND	ND	ND	ND	ND	ND	ND	ND	ND	ND
**48**	13/7/2020 – Site 2	**0.126**	ND	ND	ND	ND	ND	ND	ND	ND	ND	ND	ND	ND	ND	ND
**49**	10/8/2020 – Site 1	**0.063**	ND	ND	ND	ND	ND	ND	ND	**0.014**	ND	ND	ND	ND	ND	ND
**50**	10/8/2020 – Site 2	**0.060**	ND	ND	ND	ND	ND	ND	ND	ND	ND	ND	ND	ND	ND	ND
**51**	7/9/2020 – Site 1	**0.071**	ND	ND	**0.008**	ND	ND	ND	ND	ND	ND	ND	ND	ND	ND	ND
**52**	7/9/2020 – Site 2	**0.076**	ND	ND	**0.006**	ND	ND	ND	ND	ND	ND	ND	ND	ND	ND	ND
**53**	17/9/2020 – Site 1	**0.025**	ND	ND	**0.017**	ND	ND	ND	ND	**0.018**	ND	ND	ND	ND	ND	ND
**54**	17/9/2020 – Site 2	**0.022**	ND	ND	**0.020**	ND	ND	ND	ND	**0.042**	ND	ND	ND	ND	ND	ND

**55**	5/10/2020 – Site 1	**0.009**	ND	ND	**0.021**	ND	ND	ND	ND	ND	ND	ND	ND	ND	ND	ND
**56**	5/10/2020 – Site 2	**0.007**	ND	ND	**0.015**	ND	ND	ND	ND	ND	ND	ND	ND	ND	ND	ND
**57**	29/10/2020 – Site 1	**0.038**	ND	ND	**0.013**	ND	ND	ND	ND	**0.012**	ND	ND	ND	ND	ND	ND
**58**	29/10/2020 – Site 2	**0.030**	ND	ND	**0.010**	ND	**0.155**	ND	ND	**0.155**	ND	ND	ND	ND	ND	ND

[1] ND: Not Detected, [2] <LOQ: Values higher than limit of detection (LOD) and lower than limit of quantitation (LOQ).

	**CYN**	**ATX**	**dmMC-RR**	**MC-RR**	**NOD**	**MC-YR**	**MC-HtyR**	**dmMC-LR**	**MC-LR**	**MC-HilR**	**MC-WR**	**MC-LA**	**MC-LY**	**MC-LW**	**MC-LF**
**LOD (μg/L)**	0.001	0.001	0.002	0.001	0.002	0.004	0.007	0.004	0.004	0.006	0.006	0.003	0.006	0.004	0.005
**LOQ (μg/L)**	0.003	0.003	0.006	0.003	0.006	0.013	0.020	0.011	0.013	0.019	0.017	0.009	0.018	0.013	0.016

Table S3. Concentrations of Intracellular CTs from Lake Vegoritis (μg/L).

	**SAMPLE DETAILS**	**CYN**	**ATX**	**dmMC-RR**	**MC-RR**	**NOD**	**MC-YR**	**MC-HtyR**	**dmMC-LR**	**MC-LR**	**MC-HilR**	**MC-WR**	**MC-LA**	**MC-LY**	**MC-LW**	**MC-LF**
**1**	31/7/2018 – Site 1	ND[1]	ND	**0.003**	**0.074**	ND	**0.026**	ND	**<LOQ[2]**	**0.043**	ND	ND	ND	ND	ND	ND
**2**	31/7/2018 – Site 2	ND	ND	**<LOQ**	**0.047**	ND	**0.015**	ND	ND	**0.020**	ND	ND	ND	ND	ND	ND
**3**	6/8/2018 – Site 1	ND	ND	ND	**0.026**	ND	**0.020**	ND	**<LOQ**	**0.017**	ND	ND	ND	ND	ND	ND
**4**	6/8/2018 – Site 2	ND	ND	**<LOQ**	**0.033**	ND	**0.023**	ND	ND	**0.025**	ND	ND	ND	ND	ND	ND
**5**	21/8/2018 – Site 1	ND	ND	ND	**0.009**	ND	**<LOQ**	ND	**<LOQ**	**0.028**	ND	ND	ND	ND	ND	ND
**6**	21/8/2018 – Site 2	ND	ND	ND	**0.005**	ND	**0.007**	ND	ND	**0.006**	ND	ND	ND	ND	ND	ND
**7**	5/9/2018 – Site 1	ND	ND	ND	**<LOQ**	ND	ND	ND	ND	**0.005**	ND	ND	ND	ND	ND	ND
**8**	5/9/2018 – Site 2	ND	ND	ND	**<LOQ**	ND	ND	ND	ND	**0.003**	ND	ND	ND	ND	ND	ND
**9**	24/9/2018 – Site 1	ND	ND	ND	**<LOQ**	ND	ND	ND	ND	**<LOQ**	ND	ND	ND	ND	ND	ND
**10**	24/9/2018 – Site 2	ND	ND	ND	ND	ND	ND	ND	ND	ND	ND	ND	ND	ND	ND	ND
**11**	15/10/2018 – Site 1	ND	ND	ND	ND	ND	ND	ND	ND	ND	ND	ND	ND	ND	ND	ND

**12**	15/10/2018 – Site 2	ND	ND	ND	ND	ND	ND	ND	ND	ND	ND	ND	ND	ND	ND	ND
**13**	7/11/2018 – Site 1	ND	ND	ND	**0.027**	ND	ND	ND	ND	**0.055**	ND	ND	ND	ND	ND	ND
**14**	7/11/2018 – Site 2	ND	ND	ND	**0.011**	ND	ND	ND	ND	**0.009**	ND	ND	ND	ND	ND	ND
**15**	10/4/2019 – Site 1	ND	ND	ND	ND	ND	ND	ND	ND	ND	ND	ND	ND	ND	ND	ND
**16**	10/4/2019 – Site 2	ND	ND	ND	ND	ND	ND	ND	ND	ND	ND	ND	ND	ND	ND	ND
**17**	2/5/2019 – Site 1	ND	ND	ND	ND	ND	ND	ND	ND	ND	ND	ND	ND	ND	ND	ND
**18**	2/5/2019 – Site 2	ND	ND	ND	ND	ND	ND	ND	ND	ND	ND	ND	ND	ND	ND	ND
**19**	20/5/2019 – Site 1	ND	ND	ND	ND	ND	ND	ND	ND	**0.036**	ND	ND	ND	ND	ND	ND
**20**	20/5/2019 – Site 2	ND	ND	ND	ND	ND	ND	ND	**<LOQ**	**0.048**	ND	ND	ND	ND	ND	ND
**21**	27/5/2019 – Site 1	ND	ND	ND	ND	ND	ND	**<LOQ**	**0.055**	**0.241**	**0.027**	ND	ND	ND	ND	ND
**22**	27/5/2019 – Site 2	ND	ND	ND	ND	ND	ND	**<LOQ**	**0.046**	**0.148**	ND	ND	ND	ND	ND	ND
**23**	5/6/2019 – Site 1	ND	ND	ND	ND	ND	ND	ND	ND	ND	ND	ND	ND	ND	ND	ND
**24**	5/6/2019 – Site 2	ND	ND	ND	ND	ND	ND	ND	ND	ND	ND	ND	ND	ND	ND	ND
**25**	18/6/2019 – Site 1	ND	ND	ND	ND	ND	ND	ND	ND	ND	ND	ND	ND	ND	ND	ND
**26**	18/6/2019 – Site 2	ND	ND	ND	ND	ND	ND	ND	ND	ND	ND	ND	ND	ND	ND	ND
**27**	15/7/2019 – Site 1	ND	ND	ND	ND	ND	ND	ND	ND	**<LOQ**	ND	ND	ND	ND	ND	ND
**28**	15/7/2019 – Site 2	ND	ND	ND	ND	ND	ND	ND	ND	ND	ND	ND	ND	ND	ND	ND
**29**	5/8/2019 – Site 1	ND	ND	ND	ND	ND	ND	ND	ND	**<LOQ**	ND	ND	ND	ND	ND	ND
**30**	5/8/2019 – Site 2	ND	ND	ND	ND	ND	ND	ND	ND	**<LOQ**	ND	ND	ND	ND	ND	ND
**31**	2/9/2019 – Site 1	**0.032**	ND	ND	**<LOQ**	ND	ND	ND	ND	**<LOQ**	ND	ND	ND	ND	ND	ND

**32**	2/9/2019 – Site 2	**0.047**	ND	ND	ND	ND	ND	ND	ND	ND	ND	ND	ND	ND	ND	ND
**33**	1/10/2019 – Site 1	**0.685**	ND	ND	**0.029**	ND	ND	ND	ND	**0.105**	ND	ND	ND	ND	ND	ND
**34**	1/10/2019 – Site 2	**0.332**	ND	ND	**<LOQ**	ND	ND	ND	ND	**<LOQ**	ND	ND	ND	ND	ND	ND
**35**	10/10/2019 – Site 1	**0.567**	ND	ND	**0.009**	ND	ND	ND	ND	**0.018**	ND	ND	ND	ND	ND	ND
**36**	10/10/2019 – Site 2	**0.523**	ND	ND	**0.007**	ND	ND	ND	ND	**0.018**	ND	ND	ND	ND	ND	ND
**37**	22/10/2019 – Site 1	**0.342**	ND	ND	**<LOQ**	ND	ND	ND	ND	**<LOQ**	ND	ND	ND	ND	ND	ND
**38**	22/10/2019 – Site 2	**0.486**	ND	ND	**<LOQ**	ND	ND	ND	ND	**<LOQ**	ND	ND	ND	ND	ND	ND
**39**	19/5/2020 – Site 1	ND	ND	ND	ND	ND	ND	ND	ND	ND	ND	ND	ND	ND	ND	ND
**40**	19/5/2020 – Site 2	ND	ND	ND	ND	ND	ND	ND	ND	**<LOQ**	ND	ND	ND	ND	ND	ND
**41**	25/5/2020 – Site 1	ND	ND	ND	ND	ND	ND	ND	ND	**0.020**	ND	ND	ND	ND	ND	ND
**42**	25/5/2020 – Site 2	ND	ND	ND	ND	ND	ND	ND	ND	**0.039**	ND	ND	ND	ND	ND	ND
**43**	9/6/2020 – Site 1	ND	ND	ND	ND	ND	ND	ND	ND	ND	ND	ND	ND	ND	ND	ND
**44**	9/6/2020 – Site 2	ND	ND	ND	ND	ND	ND	ND	ND	ND	ND	ND	ND	ND	ND	ND
**45**	22/6/2020 – Site 1	ND	ND	ND	ND	ND	ND	**<LOQ**	**<LOQ**	**0.141**	**<LOQ**	ND	ND	ND	ND	ND
**46**	22/6/2020 – Site 2	ND	ND	ND	ND	ND	ND	**<LOQ**	**0.020**	**0.146**	**<LOQ**	ND	ND	ND	ND	ND
**47**	13/7/2020 – Site 1	ND	ND	ND	ND	ND	ND	ND	ND	ND	ND	ND	ND	ND	ND	ND
**48**	13/7/2020 – Site 2	ND	ND	ND	ND	ND	ND	ND	ND	ND	ND	ND	ND	ND	ND	ND
**49**	10/8/2020 – Site 1	ND	ND	ND	**<LOQ**	ND	ND	ND	ND	ND	ND	ND	ND	ND	ND	ND

**50**	10/8/2020 – Site 2	ND	ND	ND	**0.005**	ND	ND	ND	ND	ND	ND	ND	ND	ND	ND	ND
**51**	7/9/2020 – Site 1	ND	ND	**0.010**	**0.674**	ND	**0.199**	ND	ND	**0.137**	ND	ND	ND	ND	ND	ND
**52**	7/9/2020 – Site 2	**0.005**	ND	ND	**0.084**	ND	ND	ND	ND	**0.013**	ND	ND	ND	ND	ND	ND
**53**	17/9/2020 – Site 1	**0.075**	ND	**<LOQ**	**0.151**	ND	**<LOQ**	ND	ND	**0.062**	ND	ND	ND	ND	ND	ND
**54**	17/9/2020 – Site 2	**0.066**	ND	**<LOQ**	**0.080**	ND	**<LOQ**	ND	ND	**0.059**	ND	ND	ND	ND	ND	ND
**55**	5/10/2020 – Site 1	**0.070**	ND	ND	**0.240**	ND	**0.041**	ND	ND	**0.088**	ND	ND	ND	ND	ND	ND
**56**	5/10/2020 – Site 2	**0.066**	ND	ND	**0.224**	ND	**0.078**	ND	ND	**0.090**	ND	ND	ND	ND	ND	ND
**57**	29/10/2020 – Site 1	**0.016**	ND	ND	**0.159**	ND	**0.086**	ND	ND	**0.093**	ND	ND	ND	ND	ND	ND
**58**	29/10/2020 – Site 2	**0.030**	ND	**<LOQ**	**0.064**	ND	**0.044**	ND	ND	**0.040**	ND	ND	ND	ND	ND	ND

[1] ND: Not Detected, [2] <LOQ: Values higher than limit of detection (LOD) and lower than limit of quantitation (LOQ). * Limits of detection (LODs) for intracellular toxins altered based on the sample volume that passed through the filter. Indicatively, LODs were 0.001 μg/L for CYN and ATX, 0.003 μg/L for NOD while ranged from 0.001 to 0.009 μg/L for MCs when 1000 ml of water sample passed through the filter. LOQs were 0.004 μg/L for CYN and ANA-a, 0.008 μg/L for NOD and varied from 0.003 to 0.029 μg/L for MCs when 1000 ml of water sample passed through the filter.

Table S4. Concentrations of Extracellular CPs from Lake Vegoritis (μg/L).

ID	SAMPLE DETAILS	MG FR1[A]	MG FR3[A]	MG T1[A]	MG T2[A]	AER[A] 602/K139	AER[A] 298A	AEG A[A]	AP B	AP F[B]	OSC Y[B]
**1**	31/7/2018 – Site 1	ND[1]	ND	ND	ND	ND	ND	**0.015**	**0.003**	**<LOQ**	ND
**2**	31/7/2018 – Site 2	ND	ND	ND	ND	ND	ND	ND	**0.002**	**<LOQ**	ND
**3**	6/8/2018 – Site 1	ND	ND	ND	ND	ND	ND	**<LOQ**[2]	ND	ND	ND
**4**	6/8/2018 – Site 2	ND	ND	ND	ND	ND	ND	ND	ND	**<LOQ**	ND
**5**	21/8/2018 – Site 1	ND	ND	ND	ND	ND	ND	**0.022**	**0.008**	**0.003**	ND
**6**	21/8/2018 – Site 2	ND	ND	ND	ND	ND	ND	ND	**0.005**	**<LOQ**	ND
**7**	5/9/2018 – Site 1	ND	ND	ND	ND	ND	ND	ND	**<LOQ**	**<LOQ**	ND
**8**	5/9/2018 – Site 2	ND	ND	ND	ND	ND	ND	ND	**<LOQ**	**<LOQ**	ND
**9**	24/9/2018 – Site 1	ND	ND	ND	ND	ND	ND	ND	ND	ND	ND
**10**	24/9/2018 – Site 2	ND	ND	ND	ND	ND	ND	ND	ND	ND	ND
**11**	15/10/2018 – Site 1	ND	ND	ND	ND	ND	ND	ND	ND	ND	ND
**12**	15/10/2018 – Site 2	ND	ND	ND	ND	ND	ND	ND	ND	ND	ND
**13**	7/11/2018 – Site 1	na[3]	na	na	na	na	na	na	na	na	na
**14**	7/11/2018 – Site 2	na	na	na	na	na	na	na	na	na	na
**15**	10/4/2019 – Site 1	ND	ND	ND	ND	ND	ND	ND	**0.003**	**0.003**	ND
**16**	10/4/2019 – Site 2	ND	ND	ND	ND	ND	ND	ND	**0.008**	**0.008**	ND
**17**	2/5/2019 – Site 1	ND	ND	ND	ND	ND	ND	ND	ND	ND	ND
**18**	2/5/2019 – Site 2	ND	ND	ND	ND	ND	ND	ND	ND	ND	ND
**19**	20/5/2019 – Site 1	ND	ND	ND	ND	ND	ND	ND	ND	ND	ND
**20**	20/5/2019 – Site 2	ND	ND	ND	ND	ND	ND	ND	**<LOQ**	**0.005**	ND
**21**	27/5/2019 – Site 1	ND	ND	ND	ND	ND	ND	ND	**0.007**	**0.012**	**<LOQ**
**22**	27/5/2019 – Site 2	ND	ND	ND	ND	ND	ND	ND	**0.011**	**0.024**	ND
**23**	5/6/2019 – Site 1	ND	ND	ND	ND	ND	ND	ND	ND	ND	ND
**24**	5/6/2019 – Site 2	ND	ND	ND	ND	ND	ND	ND	**<LOQ**	**<LOQ**	ND
**25**	18/6/2019 – Site 1	ND	ND	ND	ND	ND	ND	ND	ND	ND	ND
**26**	18/6/2019 – Site 2	ND	ND	ND	ND	ND	ND	ND	ND	ND	ND
**27**	15/7/2019 – Site 1	ND	ND	ND	ND	ND	ND	ND	**0.004**	ND	ND
**28**	15/7/2019 – Site 2	ND	ND	ND	ND	ND	ND	ND	**0.003**	ND	ND

**29**	5/8/2019 – Site 1	ND	ND	ND	ND	ND	ND	ND	ND	ND	ND
**30**	5/8/2019 – Site 2	ND	ND	ND	ND	ND	ND	ND	**<LOQ**	**<LOQ**	ND
**31**	2/9/2019 – Site 1	ND	ND	ND	ND	ND	ND	ND	**<LOQ**	**<LOQ**	ND
**32**	2/9/2019 – Site 2	ND	ND	ND	ND	ND	ND	ND	**<LOQ**	**<LOQ**	ND
**33**	1/10/2019 – Site 1	ND	ND	ND	ND	ND	ND	ND	**<LOQ**	**0.003**	ND
**34**	1/10/2019 – Site 2	ND	ND	ND	ND	ND	ND	ND	**0.020**	**0.023**	ND
**35**	10/10/2019 – Site 1	ND	ND	ND	ND	ND	ND	ND	**<LOQ**	ND	ND
**36**	10/10/2019 – Site 2	ND	ND	ND	ND	ND	ND	ND	**<LOQ**	**<LOQ**	ND
**37**	22/10/2019 – Site 1	ND	ND	ND	ND	ND	ND	ND	**0.004**	**0.005**	ND
**38**	22/10/2019 – Site 2	ND	ND	ND	ND	ND	ND	ND	**<LOQ**	**<LOQ**	ND
**39**	19/5/2020 – Site 1	ND	ND	ND	ND	ND	ND	ND	ND	ND	ND
**40**	19/5/2020 – Site 2	ND	ND	ND	ND	ND	ND	ND	ND	ND	ND
**41**	25/5/2020 – Site 1	ND	ND	ND	ND	ND	ND	ND	**<LOQ**	ND	ND
**42**	25/5/2020 – Site 2	ND	ND	ND	ND	ND	ND	ND	**<LOQ**	ND	ND
**43**	9/6/2020 – Site 1	ND	ND	ND	ND	ND	ND	ND	ND	ND	ND
**44**	9/6/2020 – Site 2	ND	ND	ND	ND	ND	ND	ND	ND	ND	ND
**45**	22/6/2020 – Site 1	ND	ND	ND	ND	ND	ND	ND	**0.011**	**0.009**	**<LOQ**
**46**	22/6/2020 – Site 2	ND	ND	ND	ND	ND	ND	ND	**0.007**	**0.008**	ND
**47**	13/7/2020 – Site 1	ND	ND	ND	ND	ND	ND	ND	**0.004**	ND	ND
**48**	13/7/2020 – Site 2	ND	ND	ND	ND	ND	ND	ND	**0.012**	**<LOQ**	ND
**49**	10/8/2020 – Site 1	ND	ND	ND	ND	ND	ND	ND	ND	ND	ND
**50**	10/8/2020 – Site 2	ND	ND	ND	ND	ND	ND	ND	ND	ND	ND
**51**	7/9/2020 – Site 1	ND	ND	ND	ND	ND	ND	ND	**0.003**	**0.005**	ND
**52**	7/9/2020 – Site 2	ND	ND	ND	ND	ND	ND	ND	**<LOQ**	**<LOQ**	ND
**53**	17/9/2020 – Site 1	ND	ND	ND	ND	**0.154**	ND	ND	**<LOQ**	**0.005**	ND
**54**	17/9/2020 – Site 2	ND	ND	ND	ND	ND	ND	ND	**<LOQ**	**0.005**	ND
**55**	5/10/2020 – Site 1	ND	ND	ND	ND	ND	ND	ND	**0.004**	**0.005**	ND
**56**	5/10/2020 – Site 2	ND	ND	ND	ND	ND	ND	ND	**0.006**	**0.007**	ND

**57**	29/10/2020 – Site 1	ND	ND	ND	ND	ND	ND	ND	**<LOQ**	**<LOQ**	ND
**58**	29/10/2020 – Site 2	ND	ND	ND	ND	**0.042**	ND	ND	ND	ND	ND

[1] ND: Not Detected, [2] <LOQ: Values higher than limit of detection (LOD) and lower than limit of quantitation (LOQ). na: Not available data. [A] Microginin FR1, FR3, T1 & T2, Aeruginosin 602/K139 & 298A and Aeruginosamide concentrations are expressed as Microcystin-LR equivalents. [B] Anabaenopeptin F and Oscillamide Y concentrations are expressed as Anabaenopeptin B equivalents. LOD for extracellular Anabaenopeptin B was 0.001 μg/L and LOQ was 0.003 μg/L.

Table S5. Concentrations of Intracellular CPs from Lake Vegoritis (μg/L).

ID	SAMPLE DETAILS	MG FR1[A]	MG FR3[A]	MG T1[A]	MG T2[A]	AER[A] 602/K139	AER[A] 298A	AEG A[A]	AP B	AP F[B]	OSC Y[B]
**1**	31/7/2018 – Site 1	**0.064**	**0.153**	**1.16**	**0.315**	**0.048**	ND[1]	**0.068**	**0.302**	**0.117**	**0.003**
**2**	31/7/2018 – Site 2	**0.051**	**0.137**	**0.870**	**0.206**	**0.034**	ND	**0.033**	**0.160**	**0.066**	**<LOQ**
**3**	6/8/2018 – Site 1	**<LOQ[2]**	**0.028**	**0.474**	**0.105**	**0.017**	ND	**<LOQ**	**0.112**	**0.057**	**<LOQ**
**4**	6/8/2018 – Site 2	**0.026**	**0.021**	**0.218**	**0.044**	**0.030**	ND	**0.015**	**0.060**	**0.055**	**<LOQ**
**5**	21/8/2018 – Site 1	**0.031**	**0.049**	**0.336**	**0.072**	**0.047**	ND	**0.040**	**0.076**	**0.040**	**<LOQ**
**6**	21/8/2018 – Site 2	ND	**0.037**	**0.050**	**<LOQ**	**<LOQ**	ND	**<LOQ**	**0.021**	**0.022**	ND
**7**	5/9/2018 – Site 1	ND	ND	ND	ND	ND	ND	ND	**0.006**	**0.006**	ND
**8**	5/9/2018 – Site 2	ND	ND	ND	ND	ND	ND	ND	**0.005**	**0.008**	ND
**9**	24/9/2018 – Site 1	ND	ND	ND	ND	ND	ND	ND	**0.019**	**0.015**	ND
**10**	24/9/2018 – Site 2	ND	ND	ND	ND	ND	ND	ND	**0.012**	**0.010**	ND
**11**	15/10/2018 – Site 1	ND	ND	ND	ND	ND	ND	ND	**0.003**	**<LOQ**	**<LOQ**
**12**	15/10/2018 – Site 2	ND	ND	ND	ND	ND	ND	ND	**<LOQ**	ND	**0.006**
**13**	7/11/2018 – Site 1	na[3]	na	na	na	na	na	na	na	na	na
**14**	7/11/2018 – Site 2	na	na	na	na	na	na	na	na	na	na
**15**	10/4/2019 – Site 1	ND	ND	ND	ND	ND	ND	ND	**0.067**	**0.095**	**0.008**
**16**	10/4/2019 – Site 2	ND	ND	ND	ND	ND	ND	ND	**0.468**	**0.617**	**0.042**
**17**	2/5/2019 – Site 1	ND	ND	ND	ND	ND	ND	ND	**0.028**	**0.070**	**0.005**
**18**	2/5/2019 – Site 2	ND	ND	ND	ND	ND	ND	ND	**0.041**	**0.088**	**0.006**
**19**	20/5/2019 – Site 1	ND	ND	ND	ND	ND	ND	ND	**0.127**	**0.334**	**0.024**
**20**	20/5/2019 – Site 2	ND	ND	ND	ND	ND	ND	ND	**0.241**	**0.548**	**0.033**
**21**	27/5/2019 – Site 1	ND	ND	ND	ND	**<LOQ**	ND	ND	**0.574**	**1.219**	**0.078**
**22**	27/5/2019 – Site 2	ND	ND	ND	ND	**<LOQ**	ND	ND	**0.774**	**1.911**	**0.110**
**23**	5/6/2019 – Site 1	ND	ND	ND	ND	**0.049**	**0.039**	ND	**0.016**	**0.035**	**<LOQ**
**24**	5/6/2019 – Site 2	ND	ND	ND	ND	**0.128**	**<LOQ**	ND	**0.021**	**0.047**	**0.005**
**25**	18/6/2019 – Site 1	ND	ND	ND	ND	ND	ND	ND	**<LOQ**	**0.008**	ND
**26**	18/6/2019 – Site 2	ND	ND	ND	ND	ND	ND	ND	**<LOQ**	**<LOQ**	ND
**27**	15/7/2019 – Site 1	ND	ND	ND	ND	ND	ND	ND	**0.110**	**0.028**	**<LOQ**
**28**	15/7/2019 – Site 2	ND	ND	ND	ND	**<LOQ**	ND	ND	**0.098**	**0.019**	**<LOQ**

29	5/8/2019 – Site 1	ND	ND	ND	ND	ND	ND	ND	0.036	0.043	<LOQ
30	5/8/2019 – Site 2	ND	ND	ND	ND	0.067	ND	ND	0.051	0.055	<LOQ
31	2/9/2019 – Site 1	ND	ND	ND	ND	<LOQ	ND	ND	0.089	0.139	<LOQ
32	2/9/2019 – Site 2	ND	ND	ND	ND	<LOQ	ND	ND	0.147	0.199	ND
33	1/10/2019 – Site 1	ND	ND	ND	ND	0.110	ND	ND	0.238	0.256	<LOQ
34	1/10/2019 – Site 2	ND	ND	ND	ND	ND	ND	ND	0.065	0.076	ND
35	10/10/2019 – Site 1	ND	ND	ND	ND	ND	ND	ND	0.209	0.276	<LOQ
36	10/10/2019 – Site 2	ND	ND	ND	ND	ND	ND	ND	0.200	0.238	ND
37	22/10/2019 – Site 1	ND	ND	ND	ND	ND	ND	ND	0.241	0.321	ND
38	22/10/2019 – Site 2	ND	ND	ND	ND	<LOQ	ND	ND	0.307	0.454	ND
39	19/5/2020 – Site 1	ND	ND	ND	ND	ND	ND	ND	0.007	0.010	ND
40	19/5/2020 – Site 2	ND	ND	ND	ND	ND	ND	ND	0.007	0.017	ND
41	25/5/2020 – Site 1	ND	ND	ND	ND	ND	ND	ND	0.031	0.067	0.004
42	25/5/2020 – Site 2	ND	ND	ND	ND	ND	ND	ND	0.041	0.091	0.007
43	9/6/2020 – Site 1	ND	ND	ND	ND	ND	ND	ND	0.009	0.012	ND
44	9/6/2020 – Site 2	ND	ND	ND	ND	ND	ND	ND	0.006	0.008	ND
45	22/6/2020 – Site 1	ND	ND	ND	ND	ND	ND	ND	0.668	0.497	0.033
46	22/6/2020 – Site 2	ND	ND	ND	ND	ND	ND	ND	0.790	0.583	0.039
47	13/7/2020 – Site 1	<LOQ	ND	ND	ND	ND	ND	ND	0.342	0.027	<LOQ
48	13/7/2020 – Site 2	<LOQ	ND	ND	ND	0.019	ND	ND	0.813	0.054	<LOQ
49	10/8/2020 – Site 1	<LOQ	<LOQ	0.026	<LOQ	ND	ND	ND	0.005	0.007	ND
50	10/8/2020 – Site 2	<LOQ	ND	0.078	<LOQ	ND	ND	ND	0.006	0.013	ND
51	7/9/2020 – Site 1	0.593	1.68	47.0	10.7	0.282	<LOQ	0.249	0.497	1.382	0.048
52	7/9/2020 – Site 2	0.118	0.197	5.43	1.122	0.089	ND	0.023	0.085	0.150	0.005
53	17/9/2020 – Site 1	<LOQ	0.107	2.78	0.298	0.054	ND	0.036	0.074	0.187	0.005
54	17/9/2020 – Site 2	<LOQ	0.051	1.72	0.167	0.047	ND	0.020	0.048	0.135	<LOQ
55	5/10/2020 – Site 1	ND	<LOQ	0.295	0.026	0.236	<LOQ	ND	0.137	0.180	<LOQ
56	5/10/2020 – Site 2	ND	0.046	0.756	0.045	0.514	0.025	<LOQ	0.215	0.214	0.005
57	29/10/2020 – Site 1	1.33	0.917	0.895	0.177	0.119	ND	<LOQ	0.117	0.117	0.006

**58**	29/10/2020 – Site 2	**1.07**	**0.687**	**0.288**	**0.044**	**0.022**	ND	**0.019**	**0.072**	**0.069**	**<LOQ**

[1] ND: Not Detected, [2] <LOQ: Values higher than limit of detection (LOD) and lower than limit of quantitation (LOQ). na: Not available data**.** [A] Microginin FR1, FR3, T1 & T2, Aeruginosin 602/K139 & 219A and Aeruginosamide concentrations are expressed as Microcystin-LR equivalents. [B] Anabaenopeptin F and Oscillamide Y concentrations are expressed as Anabaenopeptin B equivalents. LOD for intracellular Anabaenopeptin B altered based on the sample volume that passed through the filter and was 0.001 μg/L and LOQ was 0.004 μg/L, when 1000 ml of water sample passed through the filter. Concentrations for Anabaenopeptin F and Oscillamide Y were reported based on the calibration curve of Anabaenopeptin B, while Microginin FR1, FR3, T1 & T2, Aeruginosin 602/K139 & 298A and Aeruginosamide were reported based on the calibration curve of MC-LR.
